# What can a comparative genomics approach tell us about the pathogenicity of mtDNA mutations in human populations?

**DOI:** 10.1111/eva.12851

**Published:** 2019-08-27

**Authors:** Hannah O'Keefe, Rachel Queen, Phillip Lord, Joanna L. Elson

**Affiliations:** ^1^ Institute of Genetic Medicine Newcastle University Newcastle‐upon‐Tyne UK; ^2^ School of Computing Newcastle University Newcastle‐upon‐Tyne UK; ^3^ Bioinformatics Core Facility Newcastle University Newcastle‐upon‐Tyne UK; ^4^ Centre for Human Metabonomics North‐West University Potchefstroom South Africa

**Keywords:** comparative genomics, haplogroup, mitochondrial disease, mtDNA

## Abstract

Mitochondrial disorders are heterogeneous, showing variable presentation and penetrance. Over the last three decades, our ability to recognize mitochondrial patients and diagnose these mutations, linking genotype to phenotype, has greatly improved. However, it has become increasingly clear that these strides in diagnostics have not benefited all population groups. Recent studies have demonstrated that patients from genetically understudied populations, in particular those of black African heritage, are less likely to receive a diagnosis of mtDNA disease. It has been suggested that haplogroup context might influence the presentation and penetrance of mtDNA disease; thus, the spectrum of mutations that are associated with disease in different populations. However, to date there is only one well‐established example of such an effect: the increased penetrance of two Leber's hereditary optic neuropathy mutations on a haplogroup J background. This paper conducted the most extensive investigation to date into the importance of haplogroup context on the pathogenicity of mtDNA mutations. We searched for proven human point mutations across 726 multiple sequence alignments derived from 33 non‐human species absent of disease. A total of 58 pathogenic point mutations arise in the sequences of these species. We assessed the sequence context and found evidence of population variants that could modulate the phenotypic expression of these point mutations masking the pathogenic effects seen in humans. This supports the theory that sequence context is influential in the presentation of mtDNA disease and has implications for diagnostic practices. We have shown that our current understanding of the pathogenicity of mtDNA point mutations, primarily built on studies of individuals with haplogroups HVUKTJ, will not present a complete picture. This will have the effect of creating a diagnostic inequality, whereby individuals who do not belong to these lineages are less likely to receive a genetic diagnosis.

## 1 INTRODUCTION

Mitochondria are involved in a range of cellular functions such as apoptosis and cell death, calcium buffering and the generation of ATP by oxidative phosphorylation. Mitochondrial DNA (mtDNA) is a circular chromosome comprising of ~16 kbp and encodes 13 proteins, 22 tRNAs and 2 rRNAs. Cells contain hundreds or even thousands of copies of mtDNA. In cells or tissues where the mtDNA is homoplasmic, all mitochondrial genomic sequences are the same, which is the expected state. However, it is possible for more than one mtDNA genotype to exist. When we see two mtDNA genotypes in individual cells or a tissue type, this is a state known as heteroplasmy (Tuppen, Blakely, Turnbull, & Taylor, 2010). Patients with mitochondrial disorders normally exhibit heteroplasmy, where one of the genotypes in an mtDNA species has a pathogenic mutation. Commonly, a biochemical defect will become apparent if the number of mutated sequences accounts for ≥60% of the mitochondrial genomic content, known as the threshold effect (Wallace & Chalkia, 2013). It is estimated that the prevalence of mitochondrial disorders is ~1/4,300 within the adult European population and over 2/3 of these will be due to an mtDNA mutation (Gorman et al., 2015).

Mitochondria are inherited solely down the maternal lineage and, therefore, do not undergo bi‐parental recombination (Elson et al., 2001). This has the effect that the evolution of mtDNA is defined by the emergence of distinct lineages called haplogroups. Databases such as MitoMap and Phylotree have compiled a wealth of information regarding human haplogroup lineages, mtDNA variation and disease association (Lott et al., 2013; Oven & Kayser, 2009). It should be noted that mtDNA accumulates single nucleotide variants (SNVs) at a higher rate than nuclear DNA (Song et al., 2005). This is useful for those looking at population histories as a sufficient phylogenetic signal can accumulate to study population histories (Howell, Elson, Howell, & Turnbull, 2007). However, all this variation presents challenges in the linkage of genotype to phenotype in the context of mtDNA disease.

Over the years, there has been considerable debate about the best way to link genotype to phenotype. The Yarham et al. (2011) pathogenicity scoring system is a well‐recognized, widely used system in the mitochondrial community. It is weighted towards functional studies, namely cybrid and single fibre analysis, which can clearly link genotype to phenotype (Yarham et al., 2011). MitoTip is a new tool by MitoMap designed to provide an initial pathogenicity prediction for newly identified variants. It utilizes a frequentist and evolutionary approach, taking three key observations into account: variant history and conservation; variant location; and disruption to the secondary structure (Sonney et al., 2017).

Mitochondrial disorders have been most widely studied in patients with Caucasian European haplogroups and, whilst haplogroup divergence allows the opportunity to study global migration patterns, lack of knowledge of phylogenetic diversity in non‐Caucasian and non‐European haplogroups might reduce the accuracy of clinical diagnosis in these populations (van der Westhuizen et al., 2015). Previous research in Black South African populations has shown discrepancies in the rate of diagnosis in the context of disease arising from mtDNA mutations (van der Westhuizen et al., 2015). This may be because either the pathogenic mutations or their presentation differs from those found in Caucasian Europeans. The phenotypic presentation of mitochondrial disease is also thought to differ between populations (Smuts et al., 2010) suggesting there is much still to learn about this group of diseases globally (van der Walt et al., 2012; van der Westhuizen et al., 2015). Here, additional evidence to support the importance of mitochondrial sequence context in the expression and penetrance of pathogenic mtDNA mutations is presented.

One means of exploring the impact of mtDNA sequence context is the use of sequences from non‐human species. If a non‐human animal that does not exhibit symptoms of mitochondrial disease harbours a proven point mutation associated with disease in humans, then exploring the surrounding sequence context of these species may give insight into the importance of haplogroup context in the presentation and manifestation of this group of mutations.

Previous research has suggested disease‐associated point mutations are likely to be found in non‐human species without the presence of disease. Magalhães (2005) searched a panel of consensus sequences from 12 primates and discovered a total of 46 human “disease‐associated” mutations across the mitochondrial genomes of these species (Magalhães, 2005). Similarly, Kern and Kondrashov (2004) focused on the mt‐tRNA genes and compiled single sequences from 106 species. They identified 52 pathogenic mutations across the mt‐tRNAs and proposed four mechanisms for masking pathogenic mutations that fall in the stem regions of the molecules. However, both of these studies were conducted prior to the existence of an accepted methodology to link genotype to phenotype in the context of mtDNA disease. Thus on re‐evaluation, the evidence to support a link between the variants/mutations they reported and disease in humans was often weak.

More recently, Queen, Steyn, Lord, and Elson (2017) performed a much larger study utilizing multiple sequence alignments from 33 non‐human species. This more recent study also applied an accepted inclusion criterion for the pathogenic variants (Yarham et al., 2011). Queen et al. (2017) focused on the m.3243A > G mutation which is the most prevalent mitochondrial point mutation causing disease in humans. It is a common cause of mitochondrial myopathy, encephalopathy, lactic acidosis and stroke‐like episodes (MELAS) amongst other phenotypes. Queen et al. (2017) studied the mt‐tRNA‐LEU(UUR) gene which is affected by the m.3243A > G mutation and found this pathogenic mutation was present amongst sequences from the dog (*Canis lupus familiaris*). Further exploration of the mt‐tRNA‐LEU(UUR) gene revealed two variants which change a G:U Wobble base pair and a mismatch pair to Watson‐Crick like pairs within the D‐stem. These changes to the secondary structure could mask the pathogenic effects of m.3243A > G in this species.

Four more pathogenic point mutations from mt‐tRNA‐LEU(UUR) were also identified in a selection of the non‐human species and, like m.3243A > G, evidence of potential masking variants was present (Queen et al., 2017). Subsequently, O'Keefe, Queen, Meldau, Lord, and Elson (2018) searched across the seven mitochondrial complex I protein‐encoding genes of the same 33 species. Again, pathogenic point mutations were found; however, at a much lower frequency. Three proven pathogenic point mutations were found across the seven genes of complex I, in contrast to the five point mutations in a single mt‐tRNA gene. Only one of the three mutations observed in the protein‐encoding genes m.3308T > C, exhibited its disease‐associated amino acid change as seen in humans.

Furthermore, the evidence supporting pathogenicity of this mutation is debated, particularly surrounding its role in left ventricular hypertrabeculation/noncompaction (Salas & Elson, 2012). This contrasting finding suggests that sequence context may be of less importance in the presentation and penetrance of mtDNA mutations in mt‐protein‐encoding genes (O'Keefe et al., 2018). One explanation is the differential strength of purifying selection in these genes. In murine models, changes in the protein‐encoding genes are rapidly eliminated, but changes in the mt‐tRNAs persist for many more generations (Kauppila et al., 2016; Stewart et al., 2008). This phenomenon could help maintain interactions between the mitochondrial and nuclear proteins responsible for oxidative phosphorylation. Mito‐nuclear protein interactions have been shown to influence disease manifestation in some cases (Loewen & Ganetzky, 2018), and it has been suggested that supernumerary nuclear proteins could mask certain pathogenic mutations by stabilizing the protein complexes (Mimaki, Wang, McKenzie, Thorburn, & Ryan, 2012). It might also be related to differential selective pressure on mt‐protein‐encoding genes and mt‐tRNA genes during the formation of primordial germ cells.

To expand our understanding of sequence context on the expression and penetrance of mitochondrial mutation, this study aims to continue the work of Queen et al. (2017) by identifying whether pathogenic point mutations are present in the remaining 21 tRNA genes and how the pathogenic effect seen in humans is suppressed.

## 2 MATERIALS AND METHODS

### 2.1 Multiple sequence alignment generation and quality control

Queen et al. (2017) previously compiled 2,784 mt‐tRNA sequences from 33 non‐human species GenBank records. These species were restricted to the *Chordata* phylum; each species also required a minimum of 30 complete mitochondrial sequences in GenBank as part of the selection criteria. The mt‐tRNA gene sequences from the Revised Cambridge Reference Sequence (rCRS), NC_012920.1, were added to the corresponding non‐human species FASTA files. Multiple sequence alignments were produced from the FASTA files using the ClustalW alignment algorithm (Thompson, Higgins, & Gibson, 1994). All multiple sequence alignments were quality‐controlled. Sequences > 5 nucleotides longer or shorter than the rCRS were identified with a short script which uses the Biopython AlignIO module (Cock et al., 2009; O'Keefe, 2018). These sequences were then manually inspected and either removed or trimmed accordingly. Similarly, the script was also utilized to identify sequences with ≥5 unknown bases (“N”). These sequences were then removed from the alignments.

### 2.2 Variant scoring and analysis of the MitoTip scoring system

As the genomic location of the mt‐tRNA genes can vary between species, all variants refer to the nucleotide positions within the rCRS. Equivalent positions were identified within the species alignments by their location within the individual genes. A list of human disease‐associated variants was compiled from the MitoMap database (Lott et al., 2013) [Accessed: 26‐05‐2018]. Each variant was scored for pathogenic status in accordance with the widely accepted Yarham et al. criteria (Yarham et al., 2011). FASTA sequences for each of the mt‐tRNA genes were collected from the following GenBank records: NC_012920, NC_001643, NC_001644, NC_005089, NC_001655.2, NC_006853, NC_001323, NC_002081 and NC_002082.1. ClustalW alignments of the nine sequences for each mt‐tRNA gene were performed. The Biopython AlignIO module was used to assess conservation at the site of each disease‐associated variant as part of the pathogenicity scoring criteria set forth by Yarham et al. (2011).

A search of the MitoMap database looked for each of the variants MitoTip pathogenicity predictions (Sonney et al., 2017) [Accessed: 26‐05‐2018]. The pathogenicity predictions by MitoTip were compared with the pathogenicity status of each variant derived from the scoring system devised by Yarham et al. (2011). Furthermore, the conservation index and GenBank frequencies for each pathogenic mutation were queried using the MitoMaster SNV Query search tool (Lott et al., 2013) [Accessed: 08‐06‐2018].

### 2.3 Variant search

A custom script was devised to search through the multiple sequence alignments for specific positions that correspond to the list of disease‐associated variants derived from MitoMap. The script utilizes the Biopython AlignIO module and accounts for gaps in the human reference sequence by adjusting the variant position accordingly (O'Keefe, 2018). Pathogenic mutations are termed monomorphic if they present ubiquitously across all sequences in the species in which they are identified. If the pathogenic mutations are presented in some but not all individuals from ≥1 species, they are termed polymorphic.

### 2.4 Secondary structure analysis and assessment of Watson‐Crick like base pairing

The mamit‐tRNA database was used to identify whether pathogenic mutations fell within a stem or loop of the mt‐tRNAs secondary structure and the corresponding base for each of the pathogenic mutations within the stem regions (Pütz, Dupuis, Sissler, & Florentz, 2007). The maintenance of Watson‐Crick base pairing for those within the stem regions was then assessed to identify changes at the corresponding base (O'Keefe, 2018).

### 2.5 Assessment of tertiary structure interactions

Mt‐tRNAs have nine core tertiary interactions which contribute to the folding of the cloverleaf molecule (Sprinzl, Horn, Brown, Ioudovitch, & Steinberg, 1998). Each of the pathogenic mutations and any corresponding bases involved in Watson‐Crick like pairs were assessed to determine whether they are involved in one of these tertiary interactions. If so, the multiple sequence alignments were examined to identify any variation at the corresponding sites of these interactions (O'Keefe, 2018).

### 2.6 Phylogenetic analysis and secondary structure modelling

We can investigate within‐species variability, by analysing pathogenic mutations where the minor allele arises in ≥5 individuals in any given species. Clades were determined by the collective polymorphic sites within the mt‐tRNA gene of the given species. Each clade and its frequency were then loaded into Network 4.6.1.6 for phylogenetic analysis (Bandelt, Forster, & Röhl, 1999). In addition, the sequence of each clade was modelled using the tRNA‐SE SCAN search server (Lowe & Chan, 2016). Sequence source was set to vertebrate mitochondria with search mode set as default. The models were used to identify population variants which have the potential to mask these pathogenic mutations. MitoMap was used to retrieve human mtDNA sequence records which contain the masking variants.

## 3 RESULTS

A total of 726 multiple sequence alignment files, comprising 22 mt‐tRNAs from each of the 33 species, were produced. Quality control was used to ensure the removal of poor‐quality sequences resulting in the removal of 50 Sequences across 10 species (Queen et al., 2017), see Table 1.

**Table 1:** Number of sequences per species before and after quality control

**Taxonomic order**	**Species**	**Common name**	**Number of sequences before QC**	**Number of sequences after QC**
Primates	*Pan Paniscus*	Banobo	54	54
	*Pan Troglodytes*	Central Chimpanzee	56	54
	*Pan Troglodytes Schweinfurthii*	Easter Chimpanzee	33	33
	*Pan Troglodytes Verus*	Western Chimpanzee	30	30
	*Macaca Fascicularis*	Crab‐eating Macaque	44	44
Rodentia	*Mus Musculus*	Mouse	50	50
	*Mus Musculus Domesticus*	House Mouse	59	59
	*Rattus Norvegicus*	Brown Rat	66	66
	*Myodes Glareolus*	Bank Vole	35	35
Anguilliformes	*Anguilla Anguilla*	European Eel	55	55
	*Anguilla Rostrata*	American Eel	51	51
Artiodactyla	*Bos Taurus*	Cow	275	274
	*Bos Grunniens*	Yak	83	83
	*Ovis Aries*	Sheep	94	94
Clupeiformes	*Clupea Harengus*	Atlantic Herring	100	100
Salmoniformes	*Coregonus Lavaretus*	European Whitefish	80	80
Perissodactyla	*Equus Caballus*	Horse	247	244
Galliformes	*Gallus Gallus*	Red Jungle Fowl	66	65
Carcharhiniformes	*Glyphis Glyphis*	Speartooth Shark	94	94
Cypriniformes	*Hypophthalmichthys Molitrix*	Silver Carp	30	29
	*Hypophthalmichthys Nobilis*	Bighead Carp	36	35
Cetartiodactyla	*Balenoptera Physalus*	Fin Whale	154	148
	*Bison Bison*	Bison	34	34
	*Orcinus Orca*	Killer Whale	87	87
	*Sus Scrofa*	Wild Boar	150	131
	*Syncerus Caffer*	African Buffalo	45	45
	*Tursiops Truncatus*	Common Bottlenose Dolphin	50	50
Carnivora	*Canis Lupus Familiaris*	Dog	391	389
	*Urocyon Littoralis Catalinae*	Island Fox	41	41
	*Urocyon Littoralis Clementae*	Island Fox	33	33
	*Urocyon Littoralis Santacruzae*	Island Fox	42	42
	*Ursus Arctos*	Brown Bear	74	74
	*Ursus Spelaeus*	Cave Bear (Extinct)	34	20

### 3.1 Assessment of pathogenicity and MitoTip evaluation

The Yarham et al. (2011) system was used for each variant listed on MitoMap to gain an evidence‐based assessment of the likelihood of pathogenicity. MitoTip is a new feature of the MitoMap database that provides a pathogenicity estimate for mt‐tRNA variants (Sonney et al., 2017). The results given by MitoTip were compared with the scoring results of the Yarham et al. (2011). The Yarham et al. (2011) criteria scored 113 as definitely being pathogenic mutations. MitoTip predictions matched the definitely pathogenic status of 33 of these mutations, demonstrating ~29% sensitivity. Two variants scored as probably pathogenic but were not matched by MitoTip. A further 56 of the variants scored as possibly pathogenic in accordance with Yarham et al. (2011), and only 17 were matched by MitoTip. Finally, with Yarham et al. (2011) the remaining 100 variants scored as neutral. MitoTip predicted 71 as neutral, demonstrating 71% specificity, Table 2. Overall, the results suggest that MitoTip is specific at the cost of some sensitivity.

**Table 2:** MitoTip pathogenicity prediction versus the results of the Yarham et al scoring system

		Yarham et al scoring			
		Pathogenic (113)	Probably (2)	Possibly (56)	Neutral (100)
MitoTip prediction	Pathogenic	33/113	2/2	2/56	0/100
	Probably	23/113	0/2	16/56	8/100
	Possibly	27/113	0/2	17/56	19/100
	Neutral	26/113	0/2	19/56	71/100
	No score	4/113	0/2	2/56	2/100

### 3.2 Pathogenic point mutations in non‐human species

**Figure 1:**
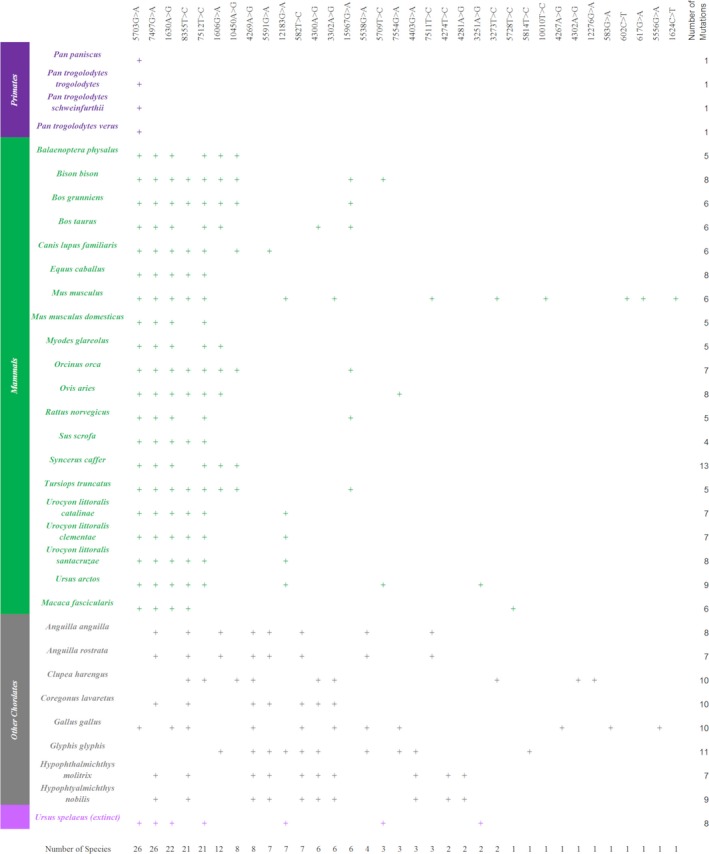
All pathogenic mutations presenting in 100% of the sequences for every species in which they are identified are classified in this study as monomorphic mutations

MitoMap lists 217 mt‐tRNA variants that have been associated with disease (Lott et al., 2013). As the assessment of pathogenicity shows, 113 of these are classified as definitely pathogenic mutations. Each of the species alignments was searched for the presence of any of these 217 variants. Across the 22 mt‐tRNA genes, 175 variants presented as either polymorphic or monomorphic changes in ≥1 species. Fifty‐eight of the changes seen are classed as definitely pathogenic mutations with 34 of these mutations being monomorphic and 24 being polymorphic, Table 3. These 58 definitely pathogenic mutations are dispersed across 19 of the 22 mt‐tRNA genes. mt‐tRNA‐GLN, mt‐tRNA‐THR and mt‐tRNA‐TYR do not harbour any proven pathogenic mutations in these species. Interestingly, ~82% of the monomorphic mutations arise in less than 10 species studied here and one, m.5703G > A, appeared in all primate species (Figure 1). This mutation has been associated with early‐onset disease presenting as muscle weakness, ophthalmoplegia and a loss of subcutaneous fat, resulting in an emaciated physique (Vives‐Bauza et al., 2003). The MitoMaster SNV query tool searches 45,494 full‐length sequences to produce a conservation index. This is the percentage of sequences which contain the same nucleotide as the rCRS at the query site (Lott et al., 2013). By consulting this, a clearer picture of conservation can be established. Table 4 demonstrates the conservation index and GenBank frequency of all 58 definitely pathogenic mutations seen in the non‐human species.

**Table 3:** (A) Mutations present in 100% of the sequences from one or more species. (B) Mutations that are polymorphic in one or more species

(A)						
Monomorphic						
Gene	Mutation	Stem	Secondary structure		Tertiary interactions	
			WC Pair	Changed in species?	Mutation	WC Pair
Ala	5591G > A	ACC	5652C > T	Some	–	–
Arg	10450A > G	T	10460T > A	Some	(13T > C‐22C > T)‐46A > G	56A > C‐19A
Asn	5703G > A	T	5687C > T	All	–	–
Asn	5728T > C	ACC	5659A > G	None	–	–
Asp	7554G > A	AC	7544C > T	None	–	–
Gly	10010T > C	D	10002A > G	None	–	–
His	12183G > A	T	12197C > T	All	(13A‐22A)‐46G > A	–
Ile	4267A > G	ACC	4326T > C	All	–	–
Ile	4269A > G	ACC	4324T > C	All	–	–
Ile	4274T > C	D	4281A > G	All	–	56C > T‐19T > C
Ile	4281A > G	D	4274T > C	All	56C > T‐19A > G	–
Ile	4300A > G	AC	4286T > C	All	–	–
Leu	3273T > C	AC	3259A > G	All	44T > C‐26C	–
Leu	3302A > G	ACC	3231T > C	All	–	–
Leu2	12276G > A	D	12288C > T	None	–	9G‐23C
Lys	8355T > C	T	8339A > G	Some	–	(25A > G‐10G)‐45A > G
Met	4403G > A	ACC	4467C > T	All	–	–
Phe	582T > C	ACC	641A > G	All	–	–
Phe	583G > A	ACC	640C > T	All	–	–
Phe	602C > T	D	586G > A	None	44A‐26C > T	(25C‐10C)‐45G
Phe	617G > A	AC	607C > T	None	–	–
Pro	15967G > A	T	15975C > T	Some	–	–
Ser	7497G > A	D	7503C > T	All	–	58A‐54C
Ser	7511T > C	ACC	7450A > G	None	–	–
Ser	7512T > C	ACC	7449A > G	All	–	–
Trp	5538G > A	AC	5552C > T	All	–	–
Val	1606G > A	ACC	1665C > T	All	–	–
Val	1624C > T	D	1611G > A	None	9A‐23C > T	(25C > T‐10G)‐45T
Val	1630A > G	AC	1638T > C	All	–	–

Monomorphic			
Gene	Mutation	Loop	Tertiary interaction
Asn	5709T > C	D	–
Cys	5814T > C	D	–
Leu	3251A > G	D	(13G‐22A > G)‐46C > T
Ile	4302A > G	VARIABLE REGION	–
Trp	5556G > A	VARIABLE REGION	(25A‐10G)‐45G > A

(B)						
Polymorphic						
Gene	Mutation	Stem	Secondary structure		Tertiary interactions	
			WC Pair	Changed in species?	Mutation	WC Pair
Ala	5628T > C	AC	5620A > G	None	–	–
Ala	5650G > A	ACC	5593C > T	None	–	–
Glu	14739G > A	ACC	14678C > T	All	–	–
Glu	14674T > C	DETERMINATOR	–	–	–	–
Ile	4284G > A	D–AC	–	–	(13G‐22G > A)‐46G > A	–
Ile	4309G > A	T	4321C > T	All	–	–
Leu	3253T > C	D	3242G > A	None	–	–
Leu	3271T > C	AC	3261A > G	All	–	–
Lys	8342G > A	T	8352C > T	None	48G > A‐15C > T	58C‐54A > T
Lys	8356T > C	T	8338A > G	Some	–	44A‐26C
Phe	642T > C	ACC	581A > G	All	–	–
Ser2	12261T > C	ACC	12210A > G	Some	55T > C‐18T	–
Ser2	12264C > T	ACC	12207G > A	Some	58C > T‐54T > C	–
Trp	5540G > A	AC	5550C > T	All	–	–

Polymorphic			
Gene	Mutation	Loop	Tertiary interaction
Asn	5693T > C	ANTI‐CD	–
Glu	14687A > G	T	(8T > A‐14A > G)‐21C
Glu	14709T > C	ANTI‐CD	–
Glu	14728T > C	D	55T > C‐19A > C
Leu	3243A > G	D	(8G‐14A > G)‐21A
Leu	3244G > A	D	–
Lys	8344A > G	T	–
Phe	622G > A	VARIABLE REGION	(13A‐22A)‐46G > A
Ser	7472A > C	VARIABLE REGION	–
Val	1644G > A	VARIABLE REGION	–

**Table 4:** Conservation index and GenBank frequency of the 58 Mutations found amongst these 33 species

Gene	Position	Mutation	Conservation index (%)	GenBank frequency
Ala	5,591	G > A	91.11	0
	5,628	T > C	95.56	88
	5,650	G > A	64.44	1
Arg	10,450	A > G	91.11	0
Asn	5,693	T > C	100	0
	5,703	G > A	8.89	0
	5,709	T > C	80	0
	5,728	T > C	86.67	1
Asp	7,554	G > A	91.11	1
Cys	5,814	T > C	75.56	128
Glu	14,674	T > C	73.33	7
	14,687	A > G	88.89	267
	14,709	T > C	95.56	1
	14,728	T > C	91.11	0
	14,739	G > A	71.11	0
Gly	10,010	T > C	100	0
His	12,183	G > A	71.11	1
Ile	4,267	A > G	93.33	0
	4,269	A > G	86.67	0
	4,274	T > C	95.56	0
	4,281	A > G	100	1
	4,284	G > A	62.22	2
	4,300	A > G	93.33	0
	4,302	A > G	97.78	0
	4,309	G > A	24.44	1
Leu	3,243	A > G	97.78	9
	3,244	G > A	95.56	6
	3,251	A > G	93.33	0
	3,253	T > C	84.44	6
	3,271	T > C	82.22	0
	3,273	T > C	97.78	0
	3,302	A > G	91.11	0
Leu2	12,276	G > A	97.78	1
Lys	8,342	G > A	62.22	0
	8,344	A > G	37.78	4
	8,355	T > C	68.89	0
	8,356	T > C	26.67	0
Met	4,403	G > A	97.78	1
Phe	582	T > C	80	0
	583	G > A	95.56	0
	602	C > T	97.78	0
	617	G > A	97.78	0
	622	G > A	93.33	0
	642	T > C	91.11	0
Pro	15,967	G > A	35.56	0
Ser	7,472	A > C	62.22	3
	7,497	G > A	8.89	1
	7,511	T > C	91.11	1
	7,512	T > C	31.11	0
Ser2	12,261	T > C	88.89	0
	12,264	C > T	71.11	0
Trp	5,538	G > A	86.67	0
	5,540	G > A	95.56	0
	5,556	G > A	93.33	0
Val	1,606	G > A	71.11	0
	1,624	C > T	97.78	0
	1,630	A > G	15.56	0
	1,644	G > A	91.11	0

Note

Derived from the MitoMaster SNV Query tool.

### 3.3 Positions of pathogenic mutations and the maintenance of Watson‐Crick base pairing

Of the 58 pathogenic mutations found in the non‐human species, 43 fall within the stem regions of their relative mt‐tRNAs and 15 within the loop regions. The 43 variants within the stem regions were assessed for a Watson‐Crick like pairing. Two of these mutations fall within nonpairing regions, the D‐AC‐stem joint and the discriminator base. One position, m.3253, is ordinarily involved in a G:U Wobble pairing, and the remaining 40 stem mutations are involved in Watson‐Crick like pairings. The single G:U Wobble base pair is transformed to a Watson‐Crick like pairing by the pathogenic mutation, m.3253G > A, and no change is seen at the corresponding base in any of the species. Watson‐Crick like pairing is maintained for 22 of the stem mutations by a change at the corresponding base across all relevant species. A further seven mutations showed a change at the corresponding base in some but not all the relevant species. Finally, 11 of the mutations did not show any change at the corresponding base, Table 3.

### 3.4 Variability in bases involved in the nine tertiary interactions

The cloverleaf structure of mt‐tRNAs undergoes a tertiary folding pattern to become an L‐shaped 3D molecule. In order to achieve this, nine long‐range folding interactions are required (Helm et al., 2000). Involvement in these tertiary interactions was noted for each of the pathogenic mutations and any corresponding bases involved in Watson‐Crick like pairing. Sixteen of the pathogenic mutations and nine of the corresponding Watson‐Crick like bases are involved in tertiary interactions. Therefore, a total of 25 tertiary interactions were explored to identify changes at the other sites. Eleven cases showed further changes; however, in six of the cases, the changes were only seen in some of the relevant species. Furthermore, 13 cases showed no changes at the additional sites, Table 3.

### 3.5 Phylogenetic analysis and secondary structure modelling

**Figure 2:**
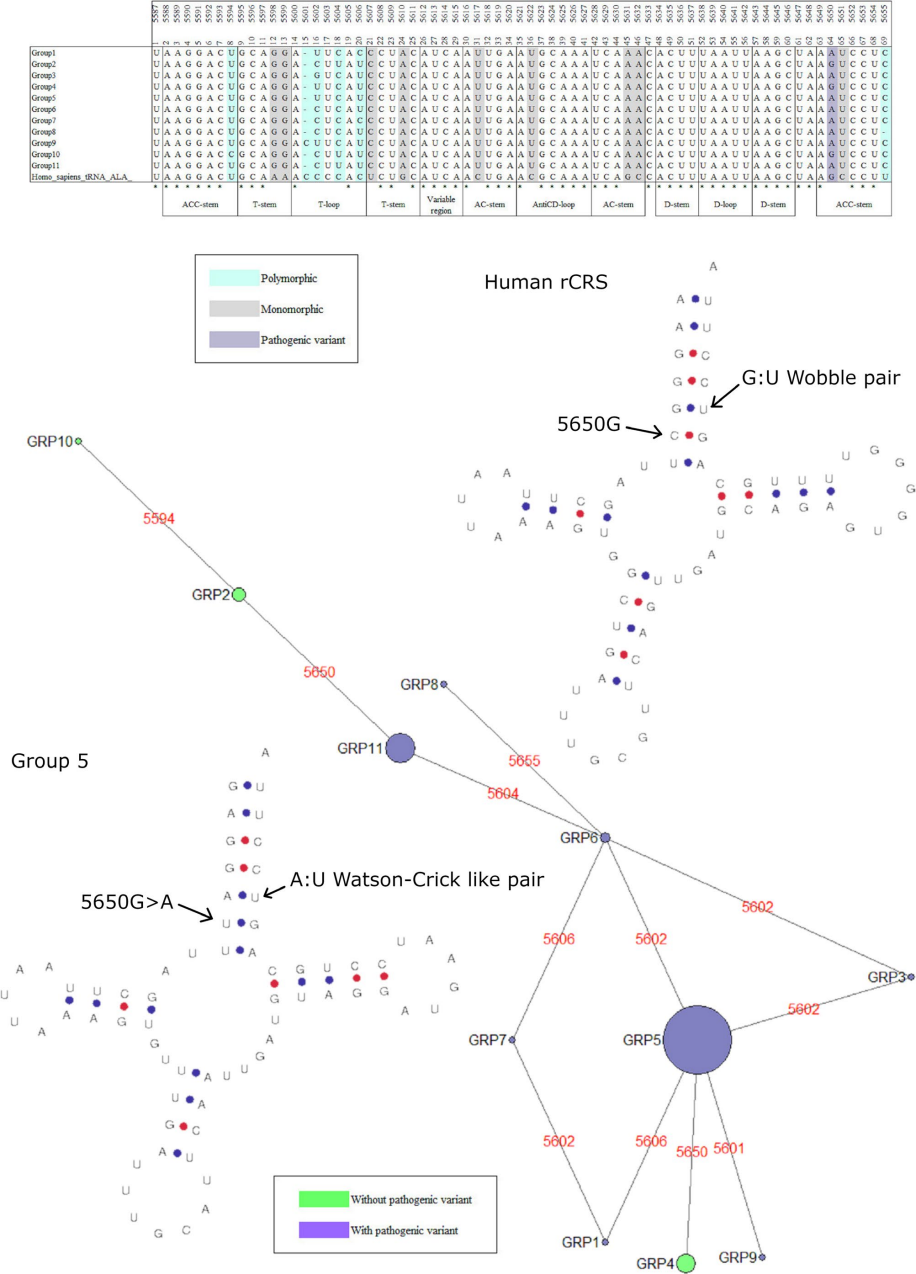
Phylogenetic analysis and secondary structure modelling of *Sus scrofa *mt‐tRNA‐Ala. Polymorphic variability in the *Sus scrofa* sequences divides the alignment into 11 clades. The phylogenetic network demonstrates the clades with and without the m.5605G > A mutation, drawn using NETWORK 4.6.0.6. As mt‐tRNA‐Ala is encoded on the heavy strand of the mitochondrial genome, all sequences and variants are denoted as the complement to the mtRNA molecule. These differences can be seen between the alignment of the clades and the secondary structure models here. Secondary structure analysis demonstrates m.5650G on the Human rCRS and its G > A change in group 5 of *Sus scrofa*. The adjoining G:U wobble pair in the rCRS and its change to an A:U Watson‐Crick like pair in *Sus scrofa* is also noted

**Figure 3:**
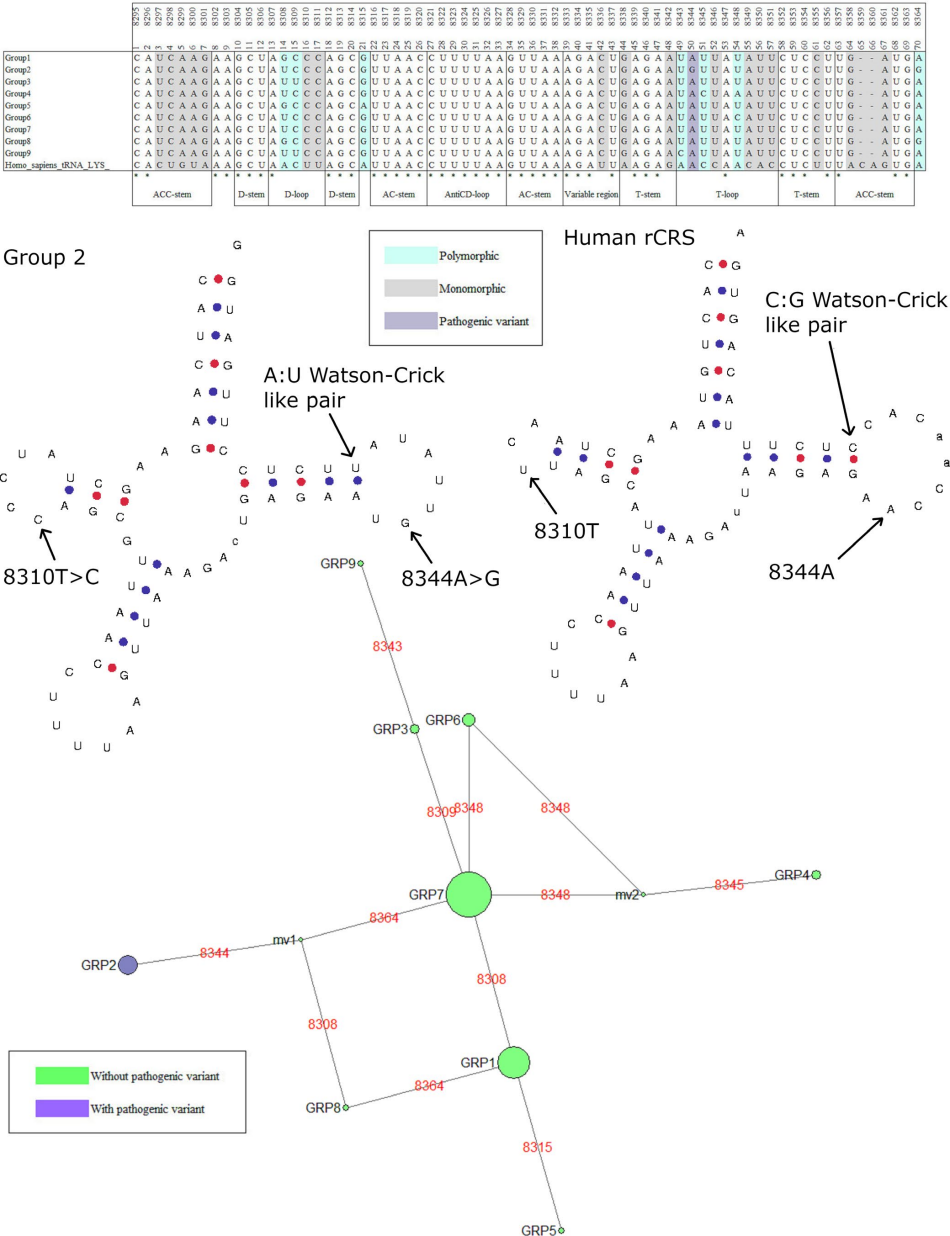
Phylogenetic analysis and secondary structure modelling of *Ovis aries* mt‐tRNA‐Lys. Nine clades were derived from polymorphic variability within the alignment. The phylogenetic network demonstrates the clades with and without the m.8344A > G mutation. Secondary structure modelling of the rCRS demonstrates m9344A in the T‐loop, the C:G Watson‐Crick like pair at the terminal of the T‐stem and m.8310T in the D‐Loop. Similarly, modelling of the *Ovis aries* group 2 sequences demonstrates m.8344A > G, a change from C:G to A:U Watson‐Crick like pairing at the terminal of the T‐stem and m.8310T > C

**Figure 4:**
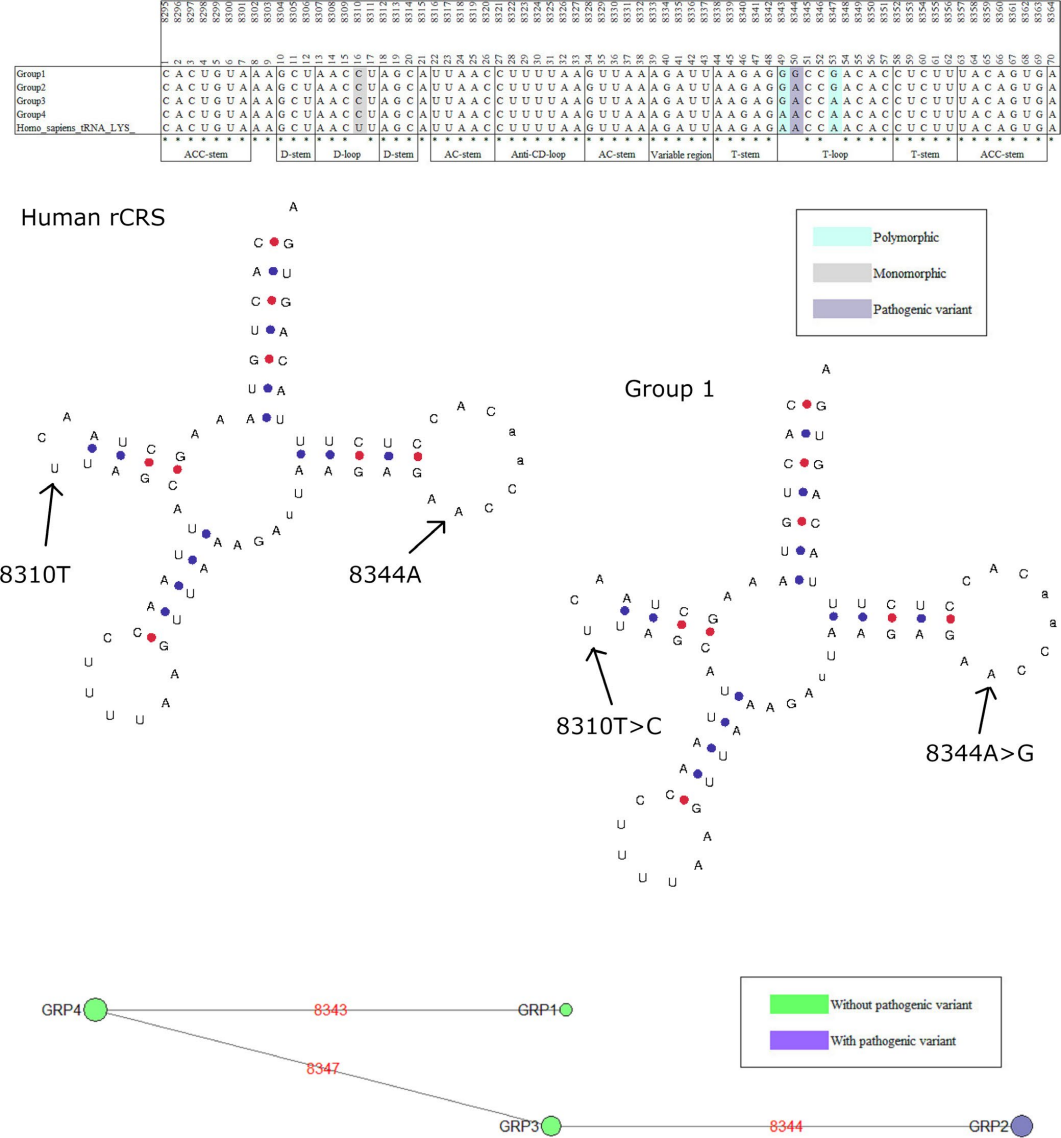
Phylogenetic analysis and secondary structure modelling of Pan troglodytes verus mt‐tRNA‐Lys. Four clades were derived from the alignment, based on polymorphic variation. The phylogenetic network demonstrates clades with and without m.8344A > G. Secondary structure modelling of the human rCRS and group 1 *Pan troglodytes *verus sequences demonstrate the m.8344A > G and m.8310T > C

**Figure 5:**
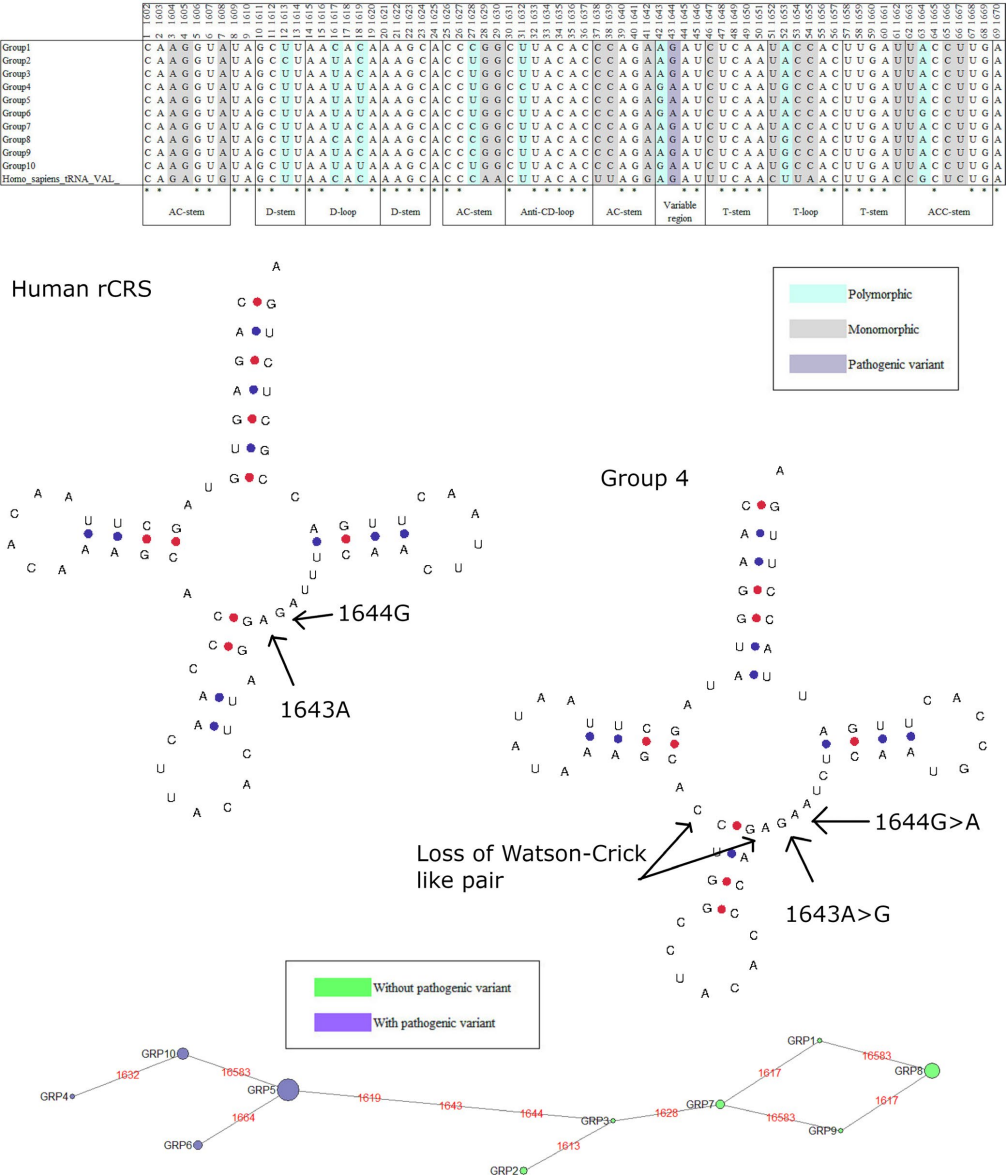
Phylogenetic analysis and secondary structure modelling of *Macaca fascicularis* mt‐tRNA‐Val. Polymorphic variability within the *M. fascicularis* alignment subdivided the sequences into 10 clades. The phylogenetic network, created with NETWORK 4.6.1.6, demonstrates clades with and without m.1644G > A. Secondary structure modelling indicates m.1644G and m.1643A in the rCRS and the m.1644G > A and m.1643A > G changes in *M. fascicularis*, along with the loss of the C:G Watson‐Crick like pairing at the terminal of the AC‐stem

Eight genes held a single pathogenic mutation where the minor allele was present in at least five individuals in any given species, Table 5. One of these was the m.3243A > G in the mt‐tRNA‐LEU(UUR) gene. As Queen et al. (2017) have already investigated this mutation and gene in detail, it was not considered for further analysis. The polymorphic mutations across the remaining seven genes were taken forward for analysis. The multiple sequence alignments of each of the species containing the polymorphic mutations were subdivided into clades according to the total polymorphic variability within the gene. Each of these clades was modelled to demonstrate the impact of the total nucleotide variability on the secondary structure of the mt‐tRNAs (Bandelt et al., 1999; Lowe & Chan, 2016). Three pathogenic mutations are of significant interest: m.5650G > A, m.8344A > G and m.1644G > A in mt‐tRNA‐Ala, mt‐tRNA‐Lys and mt‐tRNA‐Val, respectively, (Figures 2, 3, 4, 5) with data from other mutations being shown as (Figures S1–S4).

**Table 5:** Mutations arising polymorphically in one or more species

Gene	Mutation	Monomorphic	Polymorphic
Ala	5628T > C	*Anguilla anguilla*,* Anguilla rostrata*,* Clupea harangus*,* Coregonus lavaretus*	*Balaenoptera physallis (1/148)*
Ala	5650G > A	*Balaenoptera physallis*,* Bison bison*,* Bos gruniens*,* Bos taurus*,* Coregonus lavaretus*,* Glyphis glyphis*,* Myodes glareolus*,* Oricnus orca*,* Ovis aries*,* Rattus norvegicus*,* Syncerus caffer*,* Tursiops truncatus*	*Anguilla anguilla (2/55)*,* Sus scrofa (118/131)*,* Ovis aries (93/94)*
Asn	5693T > C	*–*	*Sus scrofa (1/131)*
Glu	14739G > A	*Anguilla anguilla*,* Anguilla rostrata*,* Clupea harangus*,* Macaca fascicularis*,* Mus musculus*,* Mus musculus domesticus*,* Rattus norvegicus*	*Myodes glareolus (2/35)*
Glu	14674T > C	*Ovis aries*	*Macaca fascicularis (27/44)*
Glu	14687A > G	*–*	*Canis lupus familiaris (2/389)*
Glu	14709T > C	*Clupea harangus*	*Myodes glareolus (33/35)*
Glu	14728T > C	*Gallus gallus*	*Myodes glareolus (33/35)*
Ile	4284G > A	*Gallus gallus*,* Glyphis glyphis*,* Hypophthalmichthys nobilis*,* Hypophthalmichthys molitrix*,* Oricnus orca*,* Pan paniscus*,* Pan trogolodytes trogolodytes*,* Pan trogolodytes schweinfurthii*,* Pan trogolodytes verus*,* Ursus arctos*,* Ursus spelaeus*	*Clupea harangus (2/100)*,* Coregonus lavaretus (7/80)*,* Mus musculus domesticus (58/59)*,* Syncerus caffer (1/45)*,* Tursiops truncatus (1/50)*
Ile	4309G > A	*Balaenoptera physallis*,* Bison bison*,* Bos gruniens*,* Bos taurus*,* Equus caballus*,* Macaca fascicularis*,* Mus musculus domesticus*,* Myodes glareolus*,* Oricnus orca*,* Ovis aries*,* Rattus norvegicus*,* Sus scrofa*,* Syncerus caffer*,* Tursiops truncatus*,* Urocyon litteralis clementae*,* Urocyon litteralis catalinae*,* Urocyon litteralis santacruzae*,* Ursus arctos*,* Ursus spelaeus*	*Canis lupus familiaris (386/389)*,* Clupea harangus (99/100)*,* Mus musculus (48/50)*
Leu	3253T > C	*Canis lupus familiaris*	*Macaca fascicularis (8/44)*
Leu	3271T > C	*Anguilla rostrata*,* Coregonus lavaretus*,* Gallus gallus*,* Glyphis glyphis*,* Hypophthalmichthys nobilis*,* Hypophthalmichthys molitrix*,* Mus musculus domesticus*,* Myodes glareolus*,* Rattus norvegicus*	*Anguilla anguilla (54/55)*
Leu	3243A > G	*–*	*Canis lupus familiaris (57/389)*
Leu	3244G > A	*–*	*Ursus arctos (2/74)*
Lys	8342G > A	*Ovis aries*,* Ursus arctos*	*Macaca fascicularis (44/44)*,* Sus scrofa (1/131)*
Lys	8356T > C	*Balaenoptera physallis*,* Bos taurus*,* Coregonus lavaretus*,* Equus caballus*,* Hypophthalmichthys nobilis*,* Hypophthalmichthys molitrix*,* Mus musculus domesticus*,* Myodes glareolus*,* Oricnus orca*,* Rattus norvegicus*,* Sus scrofa*,* Syncerus caffer*,* Ursus spelaeus*	*Anguilla anguilla (1/55)*
Lys	8344A > G	*Ursus arctos*	*Ovis aries (9/94)*,* Pan paniscus (3/54)*,* Pan trogolodytes verus (9/30)*,* Sus scrofa (130/131)*,* Syncerus caffer (1/45)*
Phe	642T > C	*Clupea harangus*,* Coregonus lavaretus*,* Glyphis glyphis*,* Hypophthalmichthys nobilis*,* Hypophthalmichthys molitrix*	*Gallus gallus (2/65)*
Phe	622G > A	*–*	*Balaenoptera physallis (2/148)*
Ser	7472A > C	*Balaenoptera physallis*,* Equus caballus*,* Gallus gallus*,* Oricnus orca*,* Sus scrofa*,* Tursiops truncatus*	*Ovis aries (1/94)*
Ser2	12261T > C	*Clupea harangus*,* Gallus gallus*,* Hypophthalmichthys nobilis*,* Hypophthalmichthys molitrix*	*Macaca fascicularis (27/44)*
Ser2	12264C > T	*Bos gruniens*,* Gallus gallus*,* Mus musculus domesticus*,* Myodes glareolus*,* Rattus norvegicus*	*Balaenoptera physallis (1/148)*,* Hypophthalmichthys nobilis (2/29)*,* Hypophthalmichthys molitrix (2/35)*
Trp	5540G > A	*Coregonus lavaretus*	*Oricnus orca (53/87)*
Val	1644G > A	*Glyphis glyphis*	*Macaca fascicularis (27/44)*,* Myodes glareolus (1/35)*

### 3.6 m.5650G > A mt‐tRNA‐Ala

The m.5650G > A mutation is associated with Myopathy (McFarland et al., 2008). The MitoMaster SNV query tool revealed that the conservation index for this position is 64.44% and a single GenBank record from a disease report is available, as shown in Table 4 (Annunen‐Rasila et al., 2006; Lott et al., 2013). This mutation is present in 118 of the *Sus scrofa* sequences studied here. It is also present monomorphically and as a low frequency polymorphism in other species, as shown in Table 5. The *Sus scrofa* alignment showed a further six polymorphic positions which, along with m.5650, were used to determine the clades. In addition to this, 10 positions were monomorphically divergent from the rCRS. Eleven clades were drawn from the alignment, eight of which contained m.5650G > A. The adjoining monomorphic variant, m.5651C > T, contains a base pairing of A:T transformed from G:T in all *Sus scrofa* sequences. This may stabilize the conformation in that region and act to mask the pathogenic effects of m.5650G > A (Figure 2). In GenBank, a single human mitochondrial sequence record, which belongs to haplogroup L1c, contains m.5651T.

### 3.7 m.8344A > G mt‐tRNA‐Lys

The m.8344A > G mutation is particularly interesting as this is the primary mutation for Myoclonic Epilepsy with Ragged Red Fibres (MERRF). It is thought to account for ~80% of all MERRF cases and is the second most common pathogenic point mutation in mitochondrial disorders after m.3243A > G (Lorenzoni, Scola, Kay, Silvado, & Werneck, 2014). MitoMasters SNV query tool showed the conservation index is relatively low at 37.78% (Lott et al., 2013). Only four entries are available in GenBank for this mutation, one from a disease report and three from population studies, as shown in Table 4 (Kutanan et al., 2018; Mishmar et al., 2003; Neparáczki et al., 2017; Zsurka et al., 2007). Nine individuals from *Ovis aries* and nine individuals from *Pan troglodytes verus* harbour this mutation. It is also seen as a monomorphism, near monomorphism or low frequency polymorphism in other species, Table 5. The *Ovis aries* alignments were polymorphic at another seven sites and diverged monomorphically from the rCRS at 19 sites. The alignment subdivided into nine clades, with a single clade containing m.8344A > G. Variation can be seen at ±2 positions of m.8344 and a conversion of G‐C pair to A‐T pair is seen at the final base of the T‐stem (Figure 3). The *Pan troglodytes verus* alignments diverged monomorphically from the rCRS at one site and had polymorphic variation at a further two sites, in close proximity to m.8344. This divided the alignment into four clades, with just one clade showing the m.8344A > G mutation (Figure 4). In both species, there is an m.8310T > C variant in the D‐loop. GenBank holds nine human mitochondrial sequences contain m.8310C. These sequences belong to haplogroups J1c, L2a, C, Q1a, T2b and M5a.

### 3.8 m.1644G > A mt‐tRNA‐Val

*Macaca fascicularis* exhibited the m.1644G > A mutation in 27 individuals. It is in two other species as a monomorphism and low frequency polymorphism, Table 5. This mutation is associated with MELAS (Tanji et al., 2008). The conservation index for this position is 91.11%, and no entries of this mutation are present in GenBank (Table 4; Lott et al., 2013). The presence of eight polymorphic sites alongside m.1644 meant this species alignment was subdivided into 10 clades with four clades containing m.1644G > A. There is also monomorphic divergence from the rCRS at 16 positions in *Macaca fascicularis*. The adjoining position m.1643 is one of the polymorphic sites. This site is intriguing as the polymorphism coincides with the m.1644 mutation. Between the two sites, G‐A coupling is always maintained, suggesting it may be structurally important. In GenBank, one human mitochondrial sequence from haplogroup H1u contains m.1643G. There is also a loss of the nearest Watson‐Crick like pair at the AC‐stem in all clades, which extends the length of the D‐AC‐stem joint and the variable region by one base (Figure 5).

To summarize, extensive evidence of the important of sequence context has been presented. The results are also presented as a single excel supplementary table. This table contains all the variants considered as part of this study with the results from the three different algorithms applied to score the pathogenicity, the Yarham method (Yarham et al., 2011), MitoTIP (Sonney et al., 2017) and MitoMaster SNV (Lott et al., 2013). The table also includes information on whether the variants are monomorphic and polymorphic in the named species. Importantly, information as to whether the variants are predicted to affect secondary and tertiary interactions is given, see Supplemental Tables.xlsx.

## 4 DISCUSSION

A study of South African paediatric patients showed the prevalence of known pathogenic mtDNA mutations was ~1% (van der Walt et al., 2012; van der Westhuizen et al., 2015), suggesting there is still work to be done in understanding mtDNA disease globally. We investigated the effect of mitochondrial sequence context by identifying known pathogenic mutations in species from the *Chordata* phylum. Animals have been shown to suffer from mtDNA disorders. Baranowska et al. investigated a family of Golden Retrievers with Sensory Ataxic Neuropathy. Results indicated that m.5304del on mt‐tRNA‐Tyr, equating to m.5848 in humans, had caused the disorder in the dogs (Baranowska et al., 2009). Understanding how pathogenic point mutations exist without a disease phenotype in other species may explain diagnostic variability, and lead to mechanistic insights. This paper highlights the importance of sequencing the whole mtDNA from patients especially those from less studied groups.

The secondary and tertiary folding patterns of mt‐tRNAs are well recognized. Watson‐Crick like interaction is necessary for the formation of the cloverleaf secondary structure. Queen et al. (2017) noted that known mutations falling within the stem regions of the mt‐tRNA cloverleaves often presented with a second change at the corresponding base, which should maintain the Watson‐Crick like interactions. In the current study, this phenomenon is seen with approximately half of the pathogenic mutations that arise in the stems (Table 3). It is thought that the disruption to the Watson‐Crick like bond is responsible for the manifestation of disease rather than the specific variant (McFarland, Elson, Taylor, Howell, & Turnbull, 2004). There are nine core tertiary interactions that are important for correct folding of the mt‐tRNAs into the canonical L‐shape (Helm et al., 2000). Each of the 58 pathogenic point mutations and the 41 corresponding bases in the stems were assessed for their involvement in tertiary interactions (Table 3). It is important to consider the corresponding bases in the stems as any involvement in tertiary interactions may be dominant over maintaining the secondary structure bonds. Approximately 25% of the 99 sites were involved in one of the nine interactions, just over half of which showed further changes at the other sites (Table 3). Leontis–Westhof classification states that there are 12 possible interactions between nucleotides in an RNA molecule, six *trans* and six *cis* conformations. These bonds are determined by the Hoogsteen, Sugar and Watson‐Crick edges of the nucleotides . It is thought that the 12 bonding patterns are interchangeable without disrupting the tertiary structure (Leontis & Westhof, 2001). Therefore, when a variant arises it may not be detrimental to the formation of the mt‐tRNAs L‐shape. It is also possible that changes at other sites of the tertiary interaction could mask pathogenic point mutations by exchanging bonding patterns.

Polymorphic point mutations allow the exploration of within‐species variability. Further analysis was performed for species where the minor allele arises in at least five individuals (Table 5). Three of the pathogenic point mutations were of particular of interest as they arise in species that have substantial sequence variability to define multiple clades.

Variants within mt‐tRNA‐Ala have been associated with isolated myopathy. Isolated myopathy presents as pure muscle weakness with variable age of onset (Lehmann et al., 2015). One pathogenic point mutation which causes this condition is m.5650G > A. As mt‐tRNA‐Ala is encoded on the heavy strand of the mitochondrial genome, all sequences, variants and mutations refer to the tRNA molecules complement sequence. Therefore, m.5650G > A is equal to a C > U change in the tRNA molecule itself. This mutation is polymorphic at high frequency within *Sus scrofa*, Table 5. There is monomorphic and polymorphic variability throughout the *Sus scrofa* mt‐tRNA‐Ala alignment, which could potentially contribute to masking m.5650G > A (Figure 2). One variant of interest, m.5651C > T, is present at the adjoining site in 100% of sequences. m.5651 forms part of the G:U Wobble pair that acts as the synthetase recognition site. G:U Wobble pairs give conformational flexibility within the backbone of mt‐tRNA molecules (Varani & McClain, 2000). Previous studies in *Escherichia coli* have shown that alteration of the G:U Wobble pair to an A:U pair, as seen here (Figure 2), lowers the recognition sites binding affinity but increases the stability of the backbone ([Link]). The pathogenic mutation, m.5650G > A, gives rise to a U:G Wobble pair. The angle of U:G Wobble pairs is ~2 Å different to G:U Wobble pairs and, because these sites are adjoining, the small difference in angle may be enough to abate the loss of binding affinity (Masquida & Westhof, 2000). It is plausible that this change has allowed m.5650G > A to arise without disease.

The two most commonly seen point mutations in patients with mitochondrial disorders are m.3243A > G in the D‐loop of mt‐tRNA‐Leu(URR) and m.8344A > G in the T‐loop of mt‐tRNA‐Lys. These variants are assoctiated with the MELAS and MERRF syndromes, respectively (Yarham, Elson, Blakely, McFarland, & Taylor, 2010). Myoclonic Epilepsy with Ragged Red Fibres is a highly debilitating disorder that presents primarily as ataxia, progressive spasmodic seizures and an accumulation of abnormal mitochondria under the sarcolemmal membrane of skeletal muscle fibres (Brinckmann et al., 2010; Lorenzoni et al., 2014). In all mt‐tRNAs, with the exception of mt‐tRNA‐Ser (AGY), the T‐loop is involved in long‐range tertiary interactions with the D‐loop to create the elbow of the L‐shaped structure. This elbow is important for recognition of post‐transcriptional modifiers and undergoes heavy modifications itself (Lorenz, Lünse, & Mörl, 2017). Interestingly, both m.8344A > G and m.3243A > G result in a loss of post‐transcriptional Uridine modification, τm^5^s^2^U34 and τm^5^U34, at the first wobble base of the anticodon (Yasukawa et al., 2000). A subset of *Ovis aries* and *Pan troglodytes verus* sequences contain m.8344A > G, Table 5. In both species, further variation is seen within the T‐loop and there is a T > C change at m.8310 within the D‐loop (Figures 3 and 4). Furthermore, in *Ovis aries* the T‐loop is truncated to seven bases and the G:C pair at the terminal of the T‐stem is transformed to an A:U pair (Figure 2). Similarly, Queen et al. (2017) found m.3243A > G in a selection of *Canis lupus familiaris* sequences with adaptation of a G:U wobble pair to a G:C pair at the terminal of the D‐stem. It is plausible that variation in either loop and at the terminal bases of the stems could stabilize the conformation of the elbow by reacting with local post‐transcriptional modifications. Stabilizing the tertiary structure would allow normal modification of the anticodon Uridine, masking the pathogenic phenotype in these species. Patients with m.8344G can present with deposits of brown adipose tissue around the back of the neck. These kinds of deposits are also common in neonates and hibernating species as a means of regulating body temperature. There is potential that m.8344G has arisen in response to the thermoregulatory needs of these species. Sequence context would be important in mitigating the negative phenotypes that can occur with this mutation for it to be beneficial.

The phenotypic manifestations of MELAS include neurodegeneration, myopathy, seizures, stroke‐like episodes and a build‐up of lactic acid (El‐Hattab, Adesina, Jones, & Scaglia, 2015). m.1644G > A in the variable region of mt‐tRNA‐Val has been reported as a cause of MELAS. Interestingly, a G > T change at this site is reported to cause Leigh's syndrome (Chalmers et al., 1997). This demonstrates that whilst the position of the variant may determine whether a disease arises, the particularities of the nucleotide substitution can determine the phenotypic manifestation of disease. In mt‐tRNAs, the variable region interacts with the D‐loop to form the core of the elbow and aids synthetase recognition. Post‐transcriptional modifications within this region contribute to both the stability and flexibility of the tertiary structure (Torres, Batlle, & Ribas de Pouplana, 2014). The m.1644G > A mutation is seen in a selection of *Macaca fascicularis* sequences, Table 5. In all *Macaca fascicularis* sequences, there is a loss of the Watson‐Crick like C:G pair at the terminal of the AC‐stem, truncating the stem to 4 base pairs (Figure 5). The one base elongation of the D‐AC‐stem joint and variable region, caused by this pairing loss, would alter the tertiary structure of mt‐tRNA‐Val, possibly abating the pathogenicity of m.1644G > A. Perhaps of more interest is the adjoining base, m.1643. This site presents a polymorphic A > G change in *Macaca fascicularis* sequences. These two changes, m.1644G > A and m.1643A > G, coincide with each other throughout the species, meaning an A,G or G,A couple is always seen at these positions (Figure 5). This suggests that the angle these nucleotides create may be structurally significant. By presenting the two together and maintaining this angle, the deleterious effects of m.1644G > A might be masked.

We considered the distribution of the potential masking variants, m.5651T, m.8310C and m.1643G, in human mtDNA sequences. MitoMap currently holds 46,092 full‐length human mtDNA sequences from GenBank [Accessed: August 2018]. We used MitoMap to determine which, if any, haplogroups these masking variants arise in. Both m.5651T and m.1643G arise in only a single sequence from haplogroups L1c and H1u, respectively. Interestingly, m.8310C occurs in nine sequences, most commonly in Asian and African haplogroups. This reiterates the importance of considering sequence context when looking at mtDNA mutations, particularly in understudied populations.

We noted that in these species, quite often the nearest stem pair is modified in some way (Figures 2, 3, and 5), as seen with m.3243A > G by Queen et al. (2017). Kern and Kondrashov noted also that stem pairs nearby are often modified and indirectly stabilize the pathogenic mutation where they are seen in the absence of disease (Kern & Kondrashov, 2004). Whilst these mechanisms of masking pathogenicity are speculation at this stage, it gives support to the theory that the sequence context of some haplogroup lineages can influence the manifestation of disease. It is interesting that the two most studied pathogenic mt‐tRNA point mutations, m.3243A > G and m.8344G > A, are found as polymorphic variants in these species. We now know these human *disease*‐causing mutations are population variants in other mammals (Queen et al., 2017). This provides motivation for investigating the occurrence and effects of common pathogenic point mutations in understudied populations. For a long time, it has been believed that if a variant is a haplogroup marker then it is not a candidate for being the causative variant in a patient. Perhaps we need to be open to there being some exceptions to this *rule*, especially if the variants are predicted to be deleterious (Lott et al., 2013; Sonney et al., 2017). Sequencing studies of individuals from these understudied populations would help expand our knowledge of population variation and identify whether certain variants cause deleterious effects on specific lineages.

Others have also looked at human lineages in an attempt to understand the distribution of mtDNA mutations. Wei et al looked at 30,506 complete human sequences suggesting an importance of mtDNA background, or haplogroup context in the penetrance of disease. Their data suggested disease‐causing mutations were more frequent in young sequences, or lineages (Wei, Gomez‐Duran, Hudson, & Chinnery, 2017). Similar observations have been reported in the past (Howell et al., 2007); however, other papers presented evidence to suggest all branches of the human phylogeny have been subject to the same level of purifying selection (Pereira, Soares, Radivojac, Li, & Samuels, 2011). This raises the question of the speed at which purifying selection takes place at the population level. The timeframe of this process has been a long‐standing area of debate with ramifications on the use of mtDNA as a molecular clock to study population histories (Howell et al., 2007; Howell, Howell, & Elson, 2008). The ages of the lineage in such studies are calculated using the number of differences seen in the sequences in question compared with the reference sequence the rCRS or revised Cambridge reference sequence. The reference sequence is a European sequence; thus, the age of lineages calculated by this method is dependent on the location of the sequence in question compared with the reference sequence. If the reference sequence had been at a different location different lineages would be deemed to be young/old, this has been highlighted by Behar et al. (2012). It was suggested that a change in reference sequence to a hypothesized most recent common ancestor (MRCA) of all modern humans to help avoid such confusion in the context of the “age” of a lineage. Others however argued that any such change would instigate confusion in the database that would impact negatively on the medical and forensic fields (Bandelt, Kloss‐Brandstätter, Richards, Yao, & Logan, 2014).

It is worth highlighting that compensatory nuclear DNA variants for mtDNA mutations of Complex I have also been seen in other species. The interdependent nature of mito‐nuclear proteins means nuclear variability, particularly in the supernumerary subunits, is likely to be able to resolve stability within the protein complexes (Mimaki et al., 2012). The work of Loewen and Ganetzky (2018) is an important exemplar when considering nuclear mitochondrial interactions. Their paper showed that that the phenotypic severity of a complex 1 mutation causing Leigh syndrome phenotype varies depending on the maternally inherited mitochondrial background. Leigh syndrome is a severe disorder characterized by early, progressive neurodegeneration, with both intellectual and motor difficulties, and deficient mitochondrial respiration (Lake, Compton, Rahman, & Thorburn, 2016).

For a long time, the presence of a variant as a haplogroup marker excluded it as a candidate for disease (Schon, Bonilla, & DiMauro, 1997). The data presented here and other data (Queen et al., 2017; Smuts et al., 2010) suggest out of place haplogroup markers sometimes called “private variants” should be considered as candidates and investigated using defined approaches (Yarham et al., 2011). In summary, studies such as the one presented here will allow us to gain a greater sense of the impact of mutations on tertiary structure and improve mechanistic understanding. They suggest there is clinical as well as anthropological motivation to continue to learn about mtDNA variation in populations where the mtDNA phylogeny is less well known. This knowledge might be essential to the diagnosis of disease (van der Westhuizen et al., 2015), which will be required if cutting edge therapies are to be offered to all population groups (Meldau et al., 2016). Certainly, this study reiterates that researchers and clinicians should not consider variants in isolation.

## Data Availability

The data that support the findings of this study are openly available in GenBank at https://www.ncbi.nlm.nih.gov/genbank/, reference number NC_012920, NC_001643, NC_001644, NC_005089, NC_001655.2, NC_006853, NC_001323, NC_002081, NC_002082.1.

## References

[eva12851-bib-0001] Annunen‐Rasila, J. , Finnilä, S. , Mykkänen, K. , Moilanen, J. S. , Veijola, J. , Pöyhönen, M. , … Majamaa, K. (2006). Mitochondrial DNA sequence variation and mutation rate in patients with CADASIL. Neurogenetics, 7(3), 185–194. 10.1007/s10048-006-0049-x 16807713

[eva12851-bib-0002] Bandelt, H. J. , Forster, P. , & Röhl, A. (1999). Median‐joining networks for inferring intraspecific phylogenies. Molecular Biology and Evolution, 16(1), 37–48. 10.1093/oxfordjournals.molbev.a026036 10331250

[eva12851-bib-0003] Bandelt, H. J. , Kloss-Brandstätter, A. , Richards, M. B. , Yao, Y. G. , & Logan, I. (2014). The case for the continuing use of the revised Cambridge Reference Sequence (rCRS) and the standardization of notation in human mitochondrial DNA studies. Journal of Human Genetics, 59(2), 66–77. 10.1038/jhg.2013.120 24304692

[eva12851-bib-0004] Baranowska, I. , Jäderlund, K. H. , Nennesmo, I. , Holmqvist, E. , Heidrich, N. , Larsson, N.‐G. , … Andersson, L. (2009). Sensory ataxic neuropathy in golden retriever dogs is caused by a deletion in the mitochondrial tRNATyr gene. PLOS Genetics., 5(5), e1000499 10.1371/journal.pgen.1000499 19492087 PMC2683749

[eva12851-bib-0005] Behar, D. M. , van Oven, M. , Rosset, S. , Metspalu, M. , Loogväli, E. L. , Silva, N. M. , … Villems, R. (2012). A "Copernican" reassessment of the human mitochondrial DNA tree from its root. American Journal of Human Genetics, 90(4), 675–684. 10.1016/j.ajhg.2012.03.002 22482806 PMC3322232

[eva12851-bib-0006] Brinckmann, A. , Weiss, C. , Wilbert, F. , von Moers, A. , Zwirner, A. , Stoltenburg‐Didinger, G. , … Schuelke, M. (2010). Regionalized pathology correlates with augmentation of mtDNA copy numbers in a patient with myoclonic epilepsy with ragged‐red fibers (MERRF‐syndrome). PLoS ONE, 5(10), e13513 10.1371/journal.pone.0013513 20976001 PMC2958123

[eva12851-bib-0007] Chalmers, R. M. , Lamont, P. J. , Nelson, I. , Ellison, D. W. , Thomas, N. H. , Harding, A. E. , et al. (1997). A mitochondrial DNA tRNA^Val^ point mutation associated with adult‐onset Leigh syndrome. Neurology, 49(2), 589–592. 10.1212/wnl.49.2.589 9270602

[eva12851-bib-0008] Cock, P. J. A. , Antao, T. , Chang, J. T. , Chapman, B. A. , Cox, C. J. , Dalke, A. , … de Hoon, M. J. L. (2009). Biopython: Freely available Python tools for computational molecular biology and bioinformatics. Bioinformatics, 25(11), 1422–1423. 10.1093/bioinformatics/btp163 19304878 PMC2682512

[eva12851-bib-0009] de Magalhães, J. P. (2005). Human disease‐associated mitochondrial mutations fixed in nonhuman primates. Journal of Molecular Evolution, 61(4), 491–497. 10.1007/s00239-004-0258-6 16132471

[eva12851-bib-0010] El‐Hattab, A. W. , Adesina, A. M. , Jones, J. , & Scaglia, F. (2015). MELAS syndrome: Clinical manifestations, pathogenesis, and treatment options. Molecular Genetics and Metabolism, 116(1), 4–12. 10.1016/j.ymgme.2015.06.004 26095523

[eva12851-bib-0011] Elson, J. L. , Andrews, R. M. , Chinnery, P. F. , Lightowlers, R. N. , Turnbull, D. M. , & Howell, N. (2001). Analysis of European mtDNAs for recombination. The American Journal of Human Genetics, 68(1), 145–153. 10.1086/316938 11115380 PMC1234908

[eva12851-bib-0012] fluxus‐engineering . Network phylogenetic software. Retrieved from http://www.fluxus-engineering.com/sharenet.htm

[eva12851-bib-0013] Gorman, G. S. , Schaefer, A. M. , Ng, Y. I. , Gomez, N. , Blakely, E. L. , Alston, C. L. , … McFarland, R. (2015). Prevalence of nuclear and mitochondrial DNA mutations related to adult mitochondrial disease. Annals of Neurology, 77(5), 753–759. 10.1002/ana.24362 25652200 PMC4737121

[eva12851-bib-0014] Helm, M. , Brulé, H. , Friede, D. , Giegé, R. , Pütz, D. , & Florentz, C. (2000). Search for characteristic structural features of mammalian mitochondrial tRNAs. RNA, 6(10), 1356–1379. 10.1017/S1355838200001047 11073213 PMC1370008

[eva12851-bib-0015] Howell, N. , Elson, J. L. , Howell, C. , & Turnbull, D. M. (2007). Relative rates of evolution in the coding and control regions of African mtDNAs. Molecular Biology and Evolution, 24(10), 2213–2221. 10.1093/molbev/msm147 17642471

[eva12851-bib-0016] Howell, N. , Howell, C. , & Elson, J. L. (2008). Time dependency of molecular rate estimates for mtDNA: this is not the time for wishful thinking. Heredity, 101(2), 107–108. 10.1038/hdy.2008.52 18523441

[eva12851-bib-0017] Kauppila, J. H. K. , Baines, H. L. , Bratic, A. , Simard, M.‐L. , Freyer, C. , Mourier, A. , … Stewart, J. B. (2016). A phenotype‐driven approach to generate mouse models with pathogenic mtDNA mutations causing mitochondrial disease. Cell Reports, 16(11), 2980–2990. 10.1016/j.celrep.2016.08.037 27626666 PMC5039181

[eva12851-bib-0018] Kern, A. D. , & Kondrashov, F. A. (2004). Mechanisms and convergence of compensatory evolution in mammalian mitochondrial tRNAs. Nature Genetics, 36, 1207 10.1038/ng1451 15502829

[eva12851-bib-0019] Kutanan, W. , Kampuansai, J. , Brunelli, A. , Ghirotto, S. , Pittayaporn, P. , Ruangchai, S. , … Stoneking, M. (2018). New insights from Thailand into the maternal genetic history of Mainland Southeast Asia. European Journal of Human Genetics, 26(6), 898–911. 10.1038/s41431-018-0113-7 29483671 PMC5974021

[eva12851-bib-0020] Lake, N. J. , Compton, A. G. , Rahman, S. , & Thorburn, D. R. (2016). Leigh syndrome: One disorder, more than 75 monogenic causes. Annals of Neurology, 79(2), 190–203. 10.1002/ana.24551 26506407

[eva12851-bib-0021] Lehmann, D. , Schubert, K. , Joshi, P. R. , Hardy, S. A. , Tuppen, H. A. L. , Baty, K. , … Taylor, R. W. (2015). Pathogenic mitochondrial mt‐tRNAAla variants are uniquely associated with isolated myopathy. European Journal of Human Genetics, 23, 1735 10.1038/ejhg.2015.73 25873012 PMC4519577

[eva12851-bib-0022] Leontis, N. B. , & Westhof, E. (2001). Geometric nomenclature and classification of RNA base pairs. RNA, 7(4), 499–512. 10.1017/S1355838201002515 11345429 PMC1370104

[eva12851-bib-0023] Loewen, C. A. , & Ganetzky, B. (2018). Mito‐nuclear interactions affecting lifespan and neurodegeneration in a *Drosophila* model of leigh syndrome. Genetics, 208(4), 1535–1552. 10.1534/genetics.118.300818 29496745 PMC5887147

[eva12851-bib-0024] Lorenz, C. , Lünse, C. E. , & Mörl, M. (2017). tRNA modifications: Impact on structure and thermal adaptation. Biomolecules, 7(2), 35 10.3390/biom7020035 PMC5485724 28375166

[eva12851-bib-0025] Lorenzoni, P. J. , Scola, R. H. , Kay, C. S. K. , Silvado, C. E. S. , & Werneck, L. C. (2014). When should MERRF (myoclonus epilepsy associated with ragged‐red fibers) be the diagnosis? Arquivos De Neuro‐Psiquiatria, 72, 803–811. 10.1590/0004-282X20140124 25337734

[eva12851-bib-0026] Lott, M. T. , Leipzig, J. N. , Derbeneva, O. , Xie, H. M. , Chalkia, D. , Sarmady, M. , … Wallace, D. C. (2013). mtDNA variation and analysis using MITOMAP and MITOMASTER. Current Protocols in Bioinformatics, 44(1), 23.1–26. 10.1002/0471250953.bi0123s44 PMC4257604 25489354

[eva12851-bib-0027] Lowe, T. M. , & Chan, P. P. (2016). tRNAscan‐SE On‐line: integrating search and context for analysis of transfer RNA genes. Nucleic Acids Research, 44(W1), W54–W57. 10.1093/nar/gkw413 27174935 PMC4987944

[eva12851-bib-0028] Masquida, B. , & Westhof, E. (2000). On the wobble GoU and related pairs. RNA, 6(1), 9–15. 10.1017/S1355838200992082 10668794 PMC1369889

[eva12851-bib-0029] McFarland, R. , Elson, J. L. , Taylor, R. W. , Howell, N. , & Turnbull, D. M. (2004). Assigning pathogenicity to mitochondrial tRNA mutations: When ‘definitely maybe’ is not good enough. Trends in Genetics, 20(12), 591–596. 10.1016/j.tig.2004.09.014 15522452

[eva12851-bib-0030] McFarland, R. , Swalwell, H. , Blakely, E. L. , He, L. , Groen, E. J. , Turnbull, D. M. , … Taylor, R. W. (2008). The m.5650G>A mitochondrial tRNAAla mutation is pathogenic and causes a phenotype of pure myopathy. Neuromuscular Disorders, 18(1), 63–67. 10.1016/j.nmd.2007.07.007 17825557

[eva12851-bib-0031] Meldau, S. , Riordan, G. , Van der Westhuizen, F. , Elson, J. L. , Smuts, I. , Pepper, M. S. , & Soodyall, H. (2016). Could we offer mitochondrial donation or similar assisted reproductive technology to South African patients with mitochondrial DNA disease? South African Medical Journal, 106(3), 234–236. 10.7196/SAMJ.2016.v106i3.10170 26915934

[eva12851-bib-0032] Mimaki, M. , Wang, X. , McKenzie, M. , Thorburn, D. R. , & Ryan, M. T. (2012). Understanding mitochondrial complex I assembly in health and disease. Biochimica Et Biophysica Acta (BBA) –bioenergetics, 1817(6), 851–862. 10.1016/j.bbabio.2011.08.010 21924235

[eva12851-bib-0033] Mishmar, D. , Ruiz‐Pesini, E. , Golik, P. , Macaulay, V. , Clark, A. G. , Hosseini, S. , … Wallace, D. C. (2003). Natural selection shaped regional mtDNA variation in humans. Proceedings of the National Academy of Sciences of the United States of America, 100(1), 171–176. 10.1073/pnas.0136972100 12509511 PMC140917

[eva12851-bib-0034] Neparáczki, E. , Kocsy, K. , Tóth, G. E. , Maróti, Z. , Kalmár, T. , Bihari, P. , … Török, T. (2017). Revising mtDNA haplotypes of the ancient Hungarian conquerors with next generation sequencing. PLoS ONE, 12(4), e0174886 10.1371/journal.pone.0174886 28422985 PMC5396865

[eva12851-bib-0035] O'Keefe, H. (2018). MScProjectCSC8398. Retrieved from git@gitlab.ncl.ac.uk:b3028713/test.git

[eva12851-bib-0036] O'Keefe, H. , Queen, R. A. , Meldau, S. , Lord, P. , & Elson, J. L. (2018). Haplogroup context is less important in the penetrance of mitochondrial DNA complex I mutations compared to mt‐tRNA mutations. Journal of Molecular Evolution, 86(6), 395–403. 10.1007/s00239-018-9855-7 29987491 PMC6061473

[eva12851-bib-0037] Pereira, L. , Soares, P. , Radivojac, P. , Li, B. , & Samuels, D. C. (2011). Comparing phylogeny and the predicted pathogenicity of protein variations reveals equal purifying selection across the global human mtDNA diversity. American Journal of Human Genetics, 88(4), 433–439. 10.1016/j.ajhg.2011.03.006 21457906 PMC3071914

[eva12851-bib-0038] Pütz, J. , Dupuis, B. , Sissler, M. , & Florentz, C. (2007). Mamit‐tRNA, a database of mammalian mitochondrial tRNA primary and secondary structures. RNA, 13(8), 1184–1190. 10.1261/rna.588407 17585048 PMC1924894

[eva12851-bib-0039] Queen, R. A. , Steyn, J. S. , Lord, P. , & Elson, J. L. (2017). Mitochondrial DNA sequence context in the penetrance of mitochondrial t‐RNA mutations: A study across multiple lineages with diagnostic implications. PLoS ONE, 12(11), e0187862 10.1371/journal.pone.0187862 29161289 PMC5697862

[eva12851-bib-0040] Salas, A. , & Elson, J. L. (2012). Raising doubts about the pathogenicity of mitochondrial DNA mutation m.3308T>C in left ventricular hypertraveculation/noncompaction. Cardiology, 122(2), 113–115. 22777272 10.1159/000339348

[eva12851-bib-0041] Schon, E. A. , Bonilla, E. , & DiMauro, S. (1997). Mitochondrial DNA mutations and pathogenesis. Journal of Bioenergetics and Biomembranes, 29(2), 131–149. 9239539 10.1023/a:1022685929755

[eva12851-bib-0042] Smuts, I. , Louw, R. , du Toit, H. , Klopper, B. , Mienie, L. J. , & van der Westhuizen, F. H. (2010). An overview of a cohort of South African patients with mitochondrial disorders. Journal of Inherited Metabolic Disease, 33(3), 95–104. 10.1007/s10545-009-9031-8 20135231

[eva12851-bib-0043] Song, S. , Pursell, Z. F. , Copeland, W. C. , Longley, M. J. , Kunkel, T. A. , & Mathews, C. K. (2005). DNA precursor asymmetries in mammalian tissue mitochondria and possible contribution to mutagenesis through reduced replication fidelity. Proceedings of the National Academy of Sciences of the United States of America, 102(14), 4990–4995. 10.1073/pnas.0500253102 15784738 PMC555996

[eva12851-bib-0044] Sonney, S. , Leipzig, J. , Lott, M. T. , Zhang, S. , Procaccio, V. , Wallace, D. C. , & Sondheimer, N. (2017). Predicting the pathogenicity of novel variants in mitochondrial tRNA with MitoTIP. PLoS Computational Biology, 13(12), e1005867 10.1371/journal.pcbi.1005867 29227991 PMC5739504

[eva12851-bib-0045] Sprinzl, M. , Horn, C. , Brown, M. , Ioudovitch, A. , & Steinberg, S. (1998). Compilation of tRNA sequences and sequences of tRNA genes. Nucleic Acids Research, 26(1), 148–153. 10.1093/nar/26.1.148 9399820 PMC147216

[eva12851-bib-0046] Stewart, J. B. , Freyer, C. , Elson, J. L. , Wredenberg, A. , Cansu, Z. , Trifunovic, A. , & Larsson, N.‐G. (2008). Strong Purifying Selection in Transmission of Mammalian Mitochondrial DNA. PLOS Biology, 6(1), e10 10.1371/journal.pbio.0060010 18232733 PMC2214808

[eva12851-bib-0047] Strazewski, P. , Biala, E. , Gabriel, K. , & McClain, W. H. . The relationship of thermodynamic stability at a G × U recognition site to tRNA aminoacylation specificity. RNA, 5(11), 1490–1494. 10.1017/s1355838299991586 PMC1369870 10580477

[eva12851-bib-0048] Tanji, K. , Kaufmann, P. , Naini, A. B. , Lu, J. , Parsons, T. C. , Wang, D. , … Rowland, L. P. (2008). A novel tRNAVal mitochondrial DNA mutation causing MELAS. Journal of the Neurological Sciences, 270(1), 23–27. 10.1016/j.jns.2008.01.016 18314141 PMC6195319

[eva12851-bib-0049] Thompson, J. D. , Higgins, D. G. , & Gibson, T. J. (1994). CLUSTAL W: Improving the sensitivity of progressive multiple sequence alignment through sequence weighting, position‐specific gap penalties and weight matrix choice. Nucleic Acids Research, 22(22), 4673–4680. 10.1093/nar/22.22.4673 7984417 PMC308517

[eva12851-bib-0050] Torres, A. G. , Batlle, E. , & Ribas de Pouplana, L. (2014). Role of tRNA modifications in human diseases. Trends in Molecular Medicine, 20(6), 306–314. 10.1016/j.molmed.2014.01.008 24581449

[eva12851-bib-0051] Tuppen, H. A. L. , Blakely, E. L. , Turnbull, D. M. , & Taylor, R. W. (2010). Mitochondrial DNA mutations and human disease. Biochimica Et Biophysica Acta (BBA) – Bioenergetics, 1797(2), 113–128. 10.1016/j.bbabio.2009.09.005 19761752

[eva12851-bib-0052] van der Walt, E. M. , Smuts, I. , Taylor, R. W. , Elson, J. L. , Turnbull, D. M. , Louw, R. , & van der Westhuizen, F. H. (2012). Characterization of mtDNA variation in a cohort of South African paediatric patients with mitochondrial disease. European Journal of Human Genetics, 20(6), 650–656. 10.1038/ejhg.2011.262 22258525 PMC3355259

[eva12851-bib-0053] van der Westhuizen, F. H. , Sinxadi, P. Z. , Dandara, C. , Smuts, I. , Riordan, G. , Meldau, S. , … Elson, J. L. (2015). Understanding the implications of mitochondrial DNA variation in the Health of black Southern African populations: The 2014 workshop. Human Mutation, 36(5), 569–571. 10.1002/humu.22789 25764011

[eva12851-bib-0054] van Oven, M. , & Kayser, M. (2009). Updated comprehensive phylogenetic tree of global human mitochondrial DNA variation. Human Mutation, 30(2), E386–E394. 10.1002/humu.20921 18853457

[eva12851-bib-0055] Varani, G. , & McClain, W. H. (2000). The G·U wobble base pair: A fundamental building block of RNA structure crucial to RNA function in diverse biological systems. EMBO Reports, 1(1), 18–23. 10.1093/embo-reports/kvd001 11256617 PMC1083677

[eva12851-bib-0056] Vives‐Bauza, C. , Del Toro, M. , Solano, A. , Montoya, J. , Andreu, A. L. , & Roig, M. (2003). Genotype‐phenotype correlation in the 5703G>A mutation in the tRNA(ASN) gene of mitochondrial DNA. Journal of Inherited Metabolic Disease, 26(5), 507–508. 14518831 10.1023/a:1025133629685

[eva12851-bib-0057] Wallace, D. C. , & Chalkia, D. (2013). Mitochondrial DNA genetics and the heteroplasmy conundrum in evolution and disease. Cold Spring Harbor Perspectives in Biology, 5(11), a021220 10.1101/cshperspect.a021220 24186072 PMC3809581

[eva12851-bib-0058] Wei, W. , Gomez‐Duran, A. , Hudson, G. , & Chinnery, P. F. (2017). Background sequence characteristics influence the occurrence and severity of disease‐causing mtDNA mutations. PLOS Genetics. 13(12), e1007126 10.1371/journal.pgen.eCollection 29253894 PMC5757940

[eva12851-bib-0059] Yarham, J. W. , Al‐Dosary, M. , Blakely, E. L. , Alston, C. L. , Taylor, R. W. , Elson, J. L. , & McFarland, R. (2011). A comparative analysis approach to determining the pathogenicity of mitochondrial tRNA mutations. Human Mutation, 32(11), 1319–1325. 10.1002/humu.21575 21882289

[eva12851-bib-0060] Yarham, J. W. , Elson, J. L. , Blakely, E. L. , McFarland, R. , & Taylor, R. W. (2010). Mitochondrial tRNA mutations and disease. Wiley Interdisciplinary Reviews: RNA, 1(2), 304–324. 10.1002/wrna.27 21935892

[eva12851-bib-0061] Yasukawa, T. , Suzuki, T. , Ishii, N. , Ueda, T. , Ohta, S. , & Watanabe, K. (2000). Defect in modification at the anticodon wobble nucleotide of mitochondrial tRNALys with the MERRF encephalomyopathy pathogenic mutation. FEBS Letters, 467(2), 175–178. 10.1016/S0014-5793(00)01145-5 10675533

[eva12851-bib-0062] Zsurka, G. , Hampel, K. G. , Kudina, T. , Kornblum, C. , Kraytsberg, Y. , Elger, C. E. , … Kunz, W. S. (2007). Inheritance of mitochondrial DNA recombinants in double‐heteroplasmic families: Potential implications for phylogenetic analysis. The American Journal of Human Genetics, 80(2), 298–305. 10.1086/511282 17236134 PMC1785346

