# Supplementary material for: What can a comparative genomics approach tell us about the pathogenicity of mtDNA mutations in human populations?
Source: Evol Appl. 2019 Aug 27;12(10):1912–30. doi: 10.1111/eva.12851 (PMC6824070; doi:10.1111/eva.12851)
Supplement: Supplementary file 1 [file EVA-12-1912-s001.docx]

**Supplementary Material**

**Fig S1.** Phylogenetic analysis and secondary structure modelling of *Macaca fascicularis* mt-tRNA-Glu. Polymorphic variability within *Macaca fascicularis* mt-tRNA-Glu subdivided the sequences into six clades. The phylogenetic analysis demonstrates the clades with and without m.14674A>G. Secondary structure modelling demonstrates m.14674A in the rCRS and m.14674A>G in *Macaca fascicularis.* This mutation is the discriminator base 3’ of the ACC-stem.

**Fig S2.** Phylogenetic analysis and secondary structure modelling of *Coregonus laveratus* mt-tRNA-Ile. Polymorphic variability within *Coregonus laveratus* mt-tRNA-Ile subdivided the sequences into three clades. The phylogenetic analysis demonstrates the clades with and without m.4284G>A. Secondary structure modelling demonstrates m.4284G in the rCRS and m.4284G>A in *Coregonus laveratus.* This mutation is the single base forming the AC-D stem joint.

**Fig S3.** Phylogenetic analysis and secondary structure modelling of *Macaca fascicularis* mt-tRNA-Ser(AGY). Polymorphic variability within *Macaca fascicularis* mt-tRNA-Ser(AGY) subdivided the sequences into four clades. The phylogenetic analysis demonstrates the clades with and without m.12261T>C. Secondary structure modelling demonstrates m.12261T in the rCRS and m.12261T>C in *Macaca fascicularis.* This mutation causes a Watson-Crick like A:U pair to alter to a mismatch A:C pair in the ACC-stem.

**Fig S4.** Phylogenetic analysis and secondary structure modelling of *Orcinus orca*  mt-tRNA-Trp. Polymorphic variability within *Orcinus orca*  mt-tRNA-Trp subdivided the sequences into three clades. The phylogenetic analysis demonstrates the clades with and without m.5540G>A. Secondary structure modelling demonstrates m.5540G in the rCRS and m.5540G>A in *Orcinus orca.* This mutation falls within the AC-stem and, along with a second change at the corresponding base m.5550, causes an alteration from a G:C pair to an A:U pair, maintaining a Watson-Crick like interaction.

**Fig S1.**


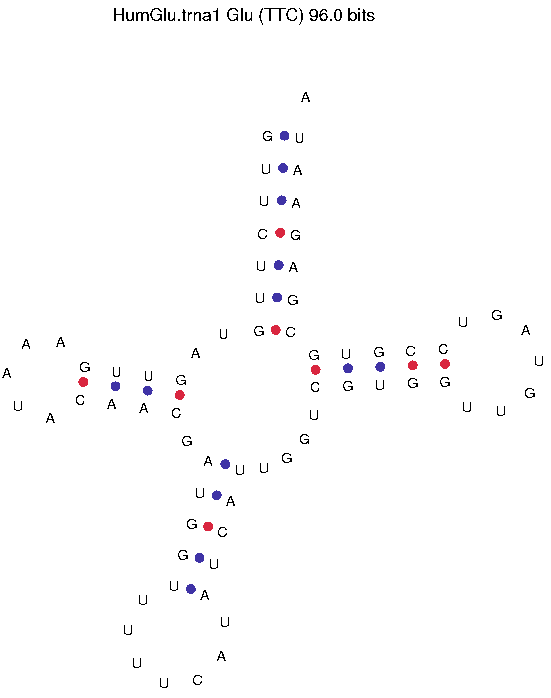


14674A

Human rCRS


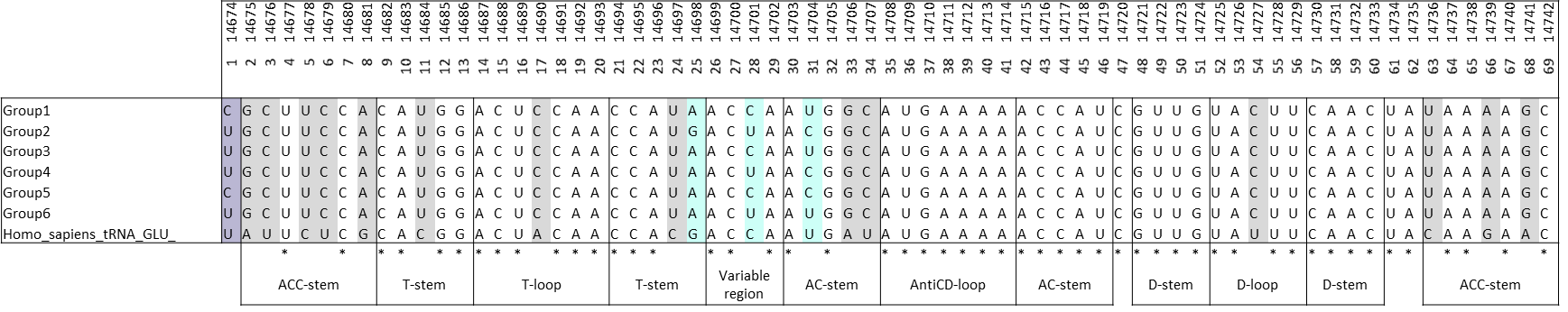

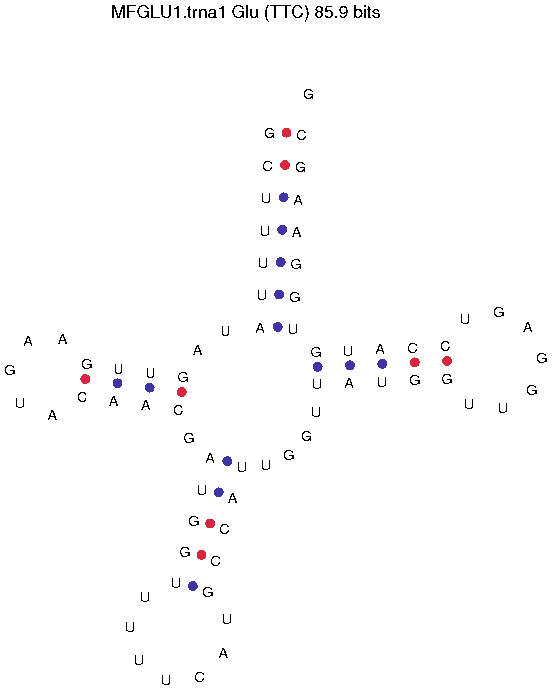


14674A>G

Group 1


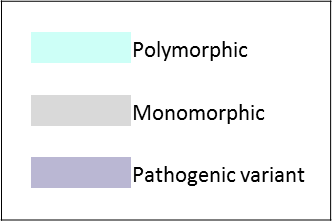

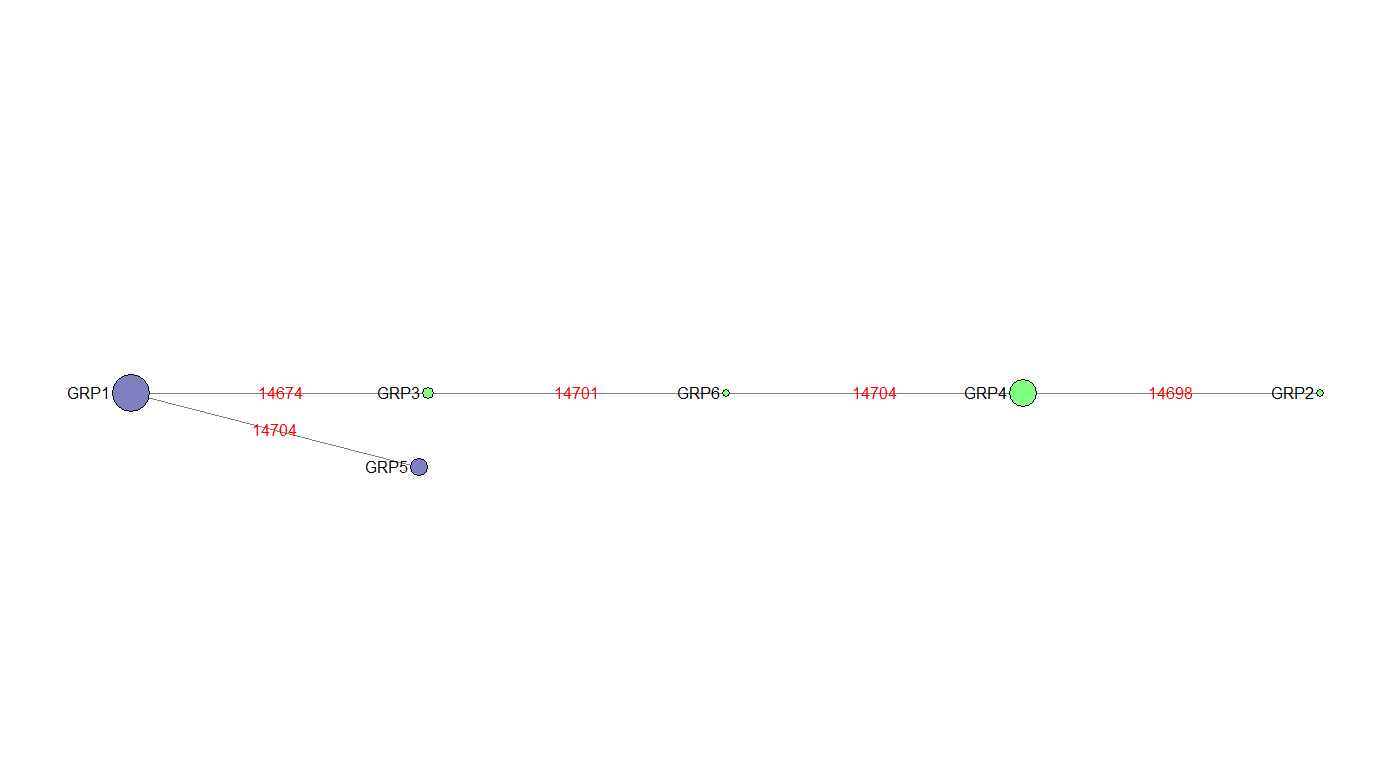

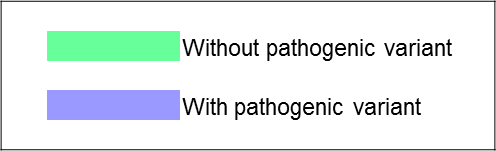


**Fig S2.**


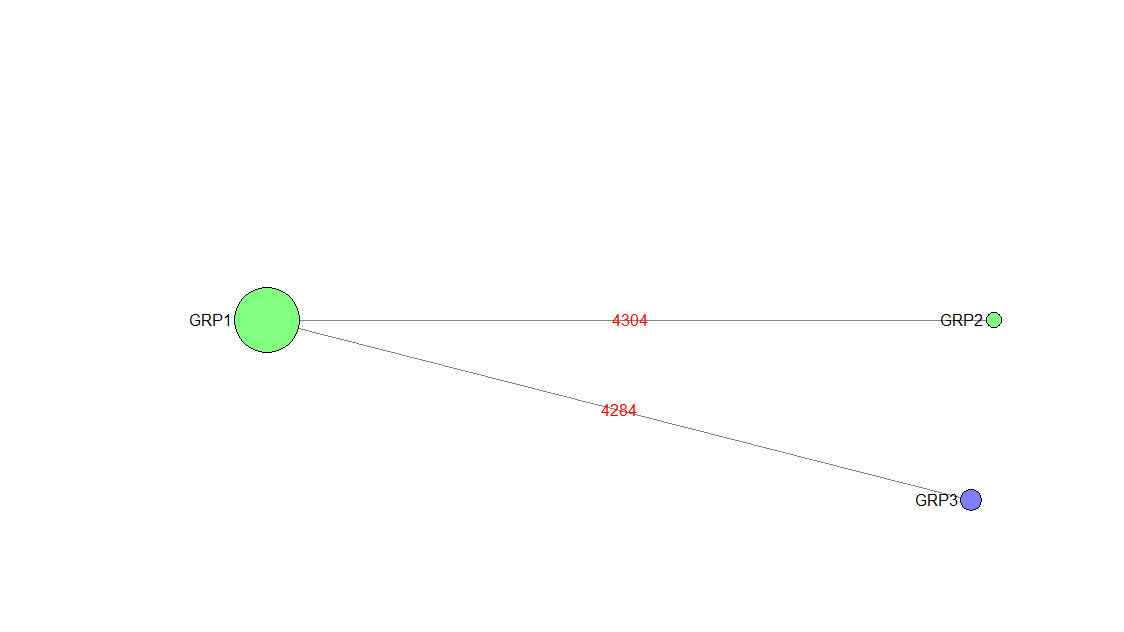

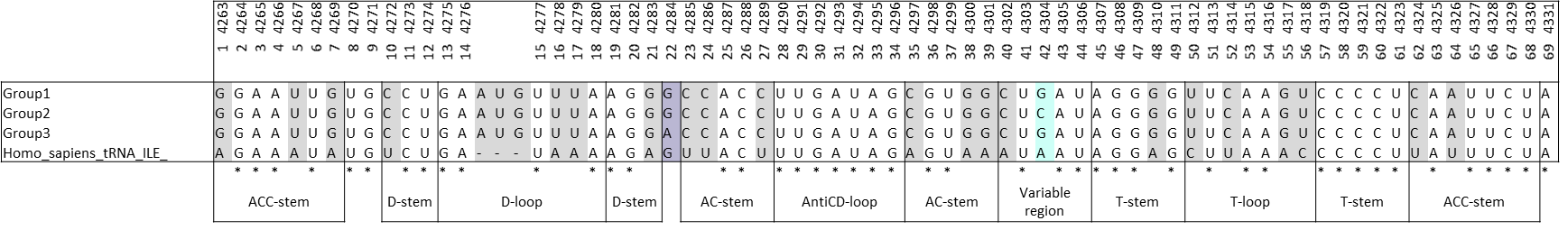

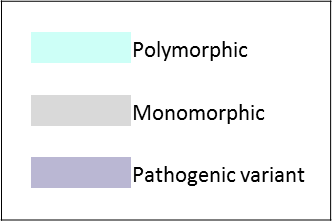

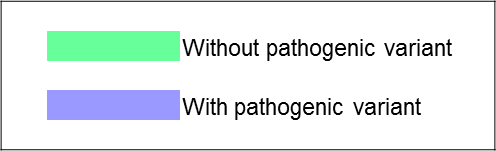

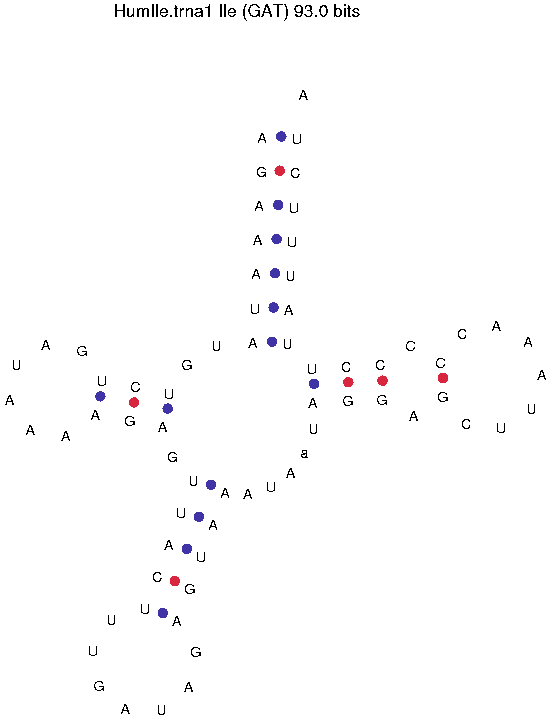


4284G

Human rCRS


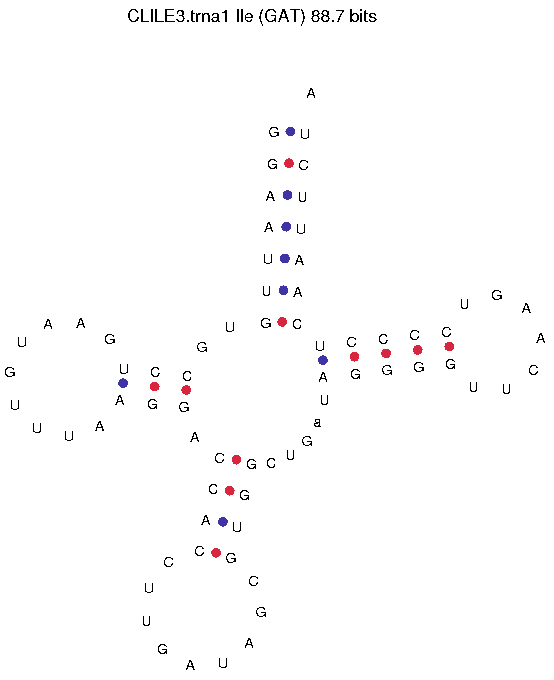


4284G>A

Group 3

**Fig S3.**


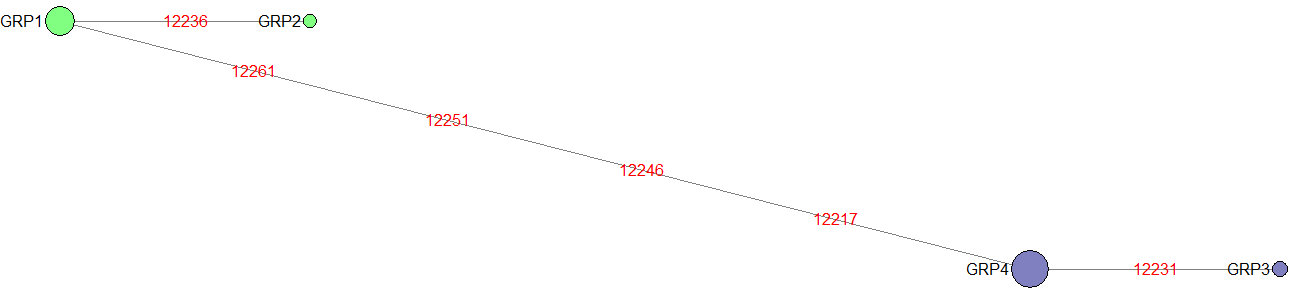

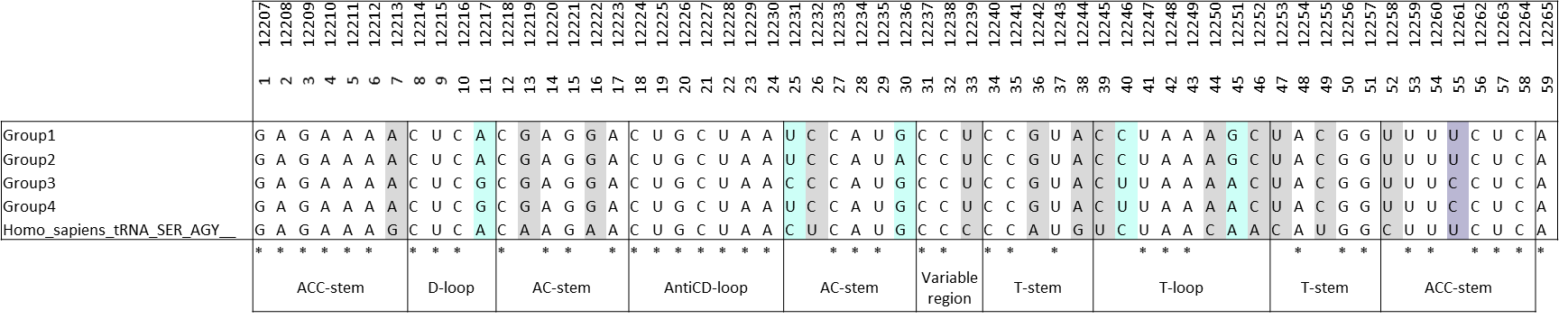

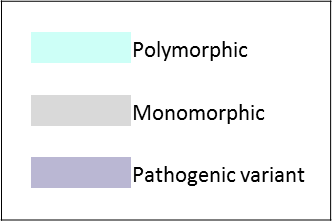

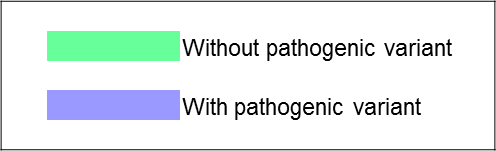

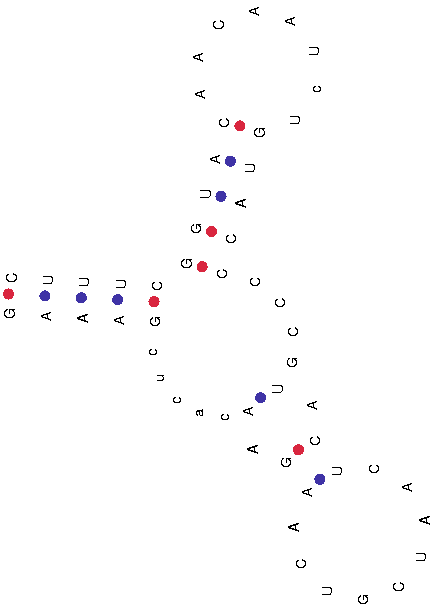


12261T

Human rCRS


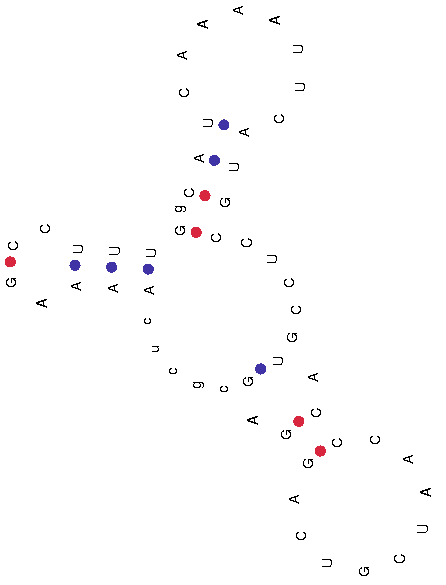


12261T>C

Group 3

**Fig S4.**


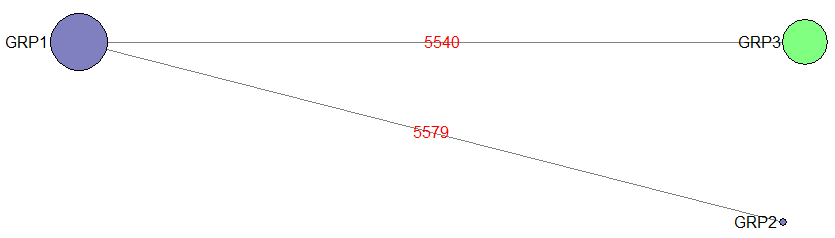

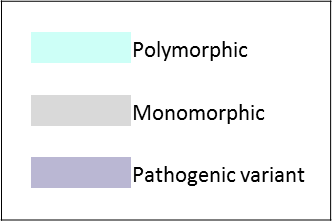

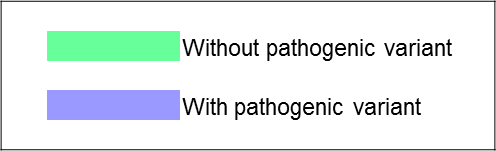

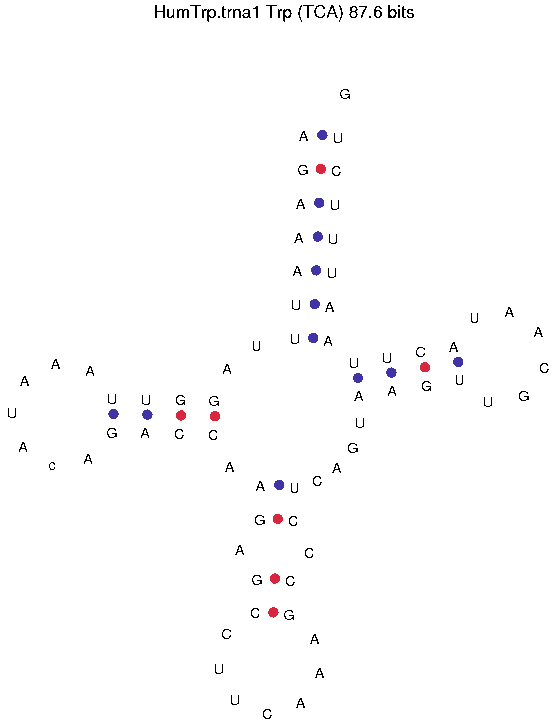


5540G

Human rCRS


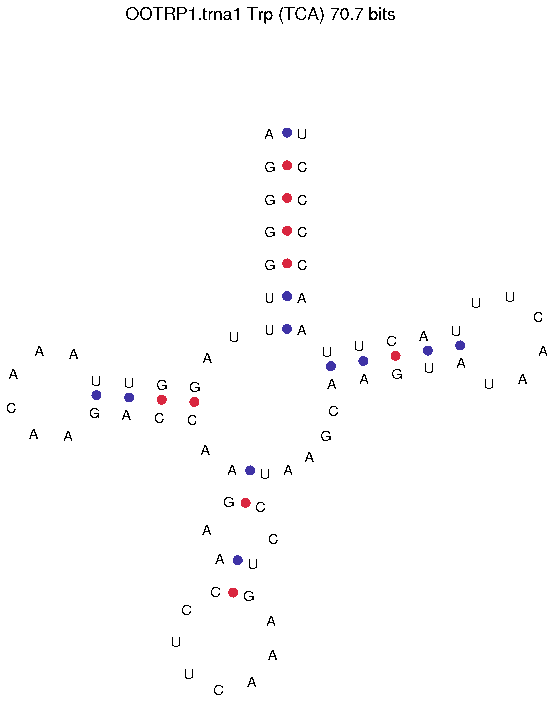


5540G>A

Group 1
